# Spatial distribution and associated factors influencing 2024 Measles-Rubella vaccination campaign coverage among children aged 9–59 months in Mainland Tanzania: A multi-level mixed-effect analysis

**DOI:** 10.1371/journal.pone.0330134

**Published:** 2026-07-21

**Authors:** Hajirani M. Msuya, Samwel Lwambura, Ibrahim Msuya, Omar Lweno, Bakari Fakih, Mwifadhi Mrisho, Hassan Tearish, Gumi Abdallah, Selemani Mmbaga, August Kuwawenaruwa, Moza Kassim, Kamaria Kassim, Farida Hassan, Joseph Mdachi, Furaha Kyesi, William Mwengee, Abdallah Mkopi

**Affiliations:** 1 Ifakara Health Institute, Dar es Salaam, Tanzania; 2 Zanzibar University, Zanzibar, Tanzania; 3 Regency Medical Centre, Paediatrics and Child Health, Dar es Salaam, Tanzania; 4 Ministry of Health, Dodoma, Tanzania; 5 World Health Organization, Tanzania Country Office, Dar es Salaam, Tanzania; CEA, FRANCE

## Abstract

**Introduction:**

Globally, measles remains a major cause of child mortality, and rubella is the leading cause of birth defects among all infectious diseases. In Mainland Tanzania, eliminating measles and rubella remains challenging due to geographical diversity, uneven healthcare facilities distribution, and socio-economic disparities across regions. Understanding spatial patterns and associated determinants of vaccination coverage is essential for improving campaign effectiveness. This study aimed to explore the spatial distribution and associated factors of measles-rubella campaign coverage among children aged 9–59 months in Mainland Tanzania.

**Methods:**

A cross-sectional survey was conducted following the implementation of the February 2024 measles-rubella (MR) vaccination campaign to assess the spatial distribution and factors influencing MR vaccination coverage among children aged 9–59 months in Mainland Tanzania. Spatial autocorrelation was evaluated using Moran’s I to detect clustering, while Local Indicators of Spatial Association (LISA) was used to identify High-High and Low-Low clusters. Hotspot and cold-spot analyses were performed using Getis–Ord Gi* statistics at the 95% confidence level**,** consistent with standard epidemiological reporting practices, to identify statistically significant spatial clusters. To identify factors associated with the campaign coverage, we used a multivariable logistic regression model.

**Results:**

The study included 16,703 children, of whom 81.5% received the MR vaccine during the campaign. Vaccination coverage varied notably between regions, with Tabora and Pwani having a low coverage rate of 58.8% (95% CI: 51.3%−65.9%) and 61.0% (95% CI: 45.0%−75.0%) respectively. Njombe and Mbeya demonstrated high MR vaccination coverage of 97.4% (95% CI: 90.5–99.3%) and 95.6% (95% CI: 90.9–97.9%), respectively. The household wealth quintile and place of residence, caregiver’s education, caregiver’s age, and their marital status were associated with receiving MR vaccination during the campaign among children aged 9–59 months in Mainland Tanzania. Spatial distribution revealed significant clustering of vaccination coverage (Moran’s I = 0.34, p < 0.01). The LISA identified two distinct categories of clusters: High-High Clusters (high-coverage) and Low-Low Clusters (low-coverage). High-High Clusters, which indicate regions with high MR vaccine rates surrounded by similar neighbors, are concentrated in regions such as Njombe and parts of Mbeya, whereas Low-Low Clusters, representing regions with low MR coverage, are found in areas like Tabora, Katavi, Dar es Salaam and Pwani. The Getis-Ord Gi* hotspot analysis shows significant clustering, with high-confidence hotspots in the southern highlands (Njombe and Mbeya) and notable cold spots in western Tanzania (Tabora and Katavi) and parts of eastern Tanzania.

**Conclusion:**

This study demonstrates substantial spatial heterogeneity in measles–rubella vaccination coverage across Mainland Tanzania, with persistent geographic inequities driven by socio-economic and demographic factors. These findings demonstrate how integrating geospatial insights with equity-focused planning can support precision public health planning, enabling targeted interventions to close coverage gaps and accelerate progress toward measles and rubella elimination.

## Background

Measles, a highly contagious viral disease, remains a leading cause of vaccine-preventable deaths among young children globally, despite the availability of an effective vaccine [1]. The measles vaccine has significantly reduced cases and deaths worldwide, but coverage gaps persist, particularly in sub-Saharan Africa, where infrastructure, healthcare access, and socio-economic factors hinder vaccination efforts [2,3]. Additionally, rubella infection during pregnancy can lead to congenital rubella syndrome (CRS), resulting in significant disability and health costs [4]. Both diseases remain critical public health challenges, with measles and rubella elimination efforts often combined in a two-dose vaccination approach as recommended by the World Health Organization (WHO) [5].

In Tanzania, efforts to eliminate measles and rubella have led to the implementation of various vaccination campaigns targeting children under five years old [6,7]. These campaigns are designed to close immunity gaps and interrupt virus transmission, particularly in populations with historically low routine immunization coverage. However, achieving and sustaining optimal MR vaccination coverage remains challenging due to Tanzania’s geographical diversity, uneven distribution of healthcare facilities, and marked regional socio-economic disparities [8]. Prior assessments of MR vaccination coverage in the year 2019 have shown substantial variations across the country, at the national level, coverage of MR was at 88.2%, with MR coverage varying across regions from 69.0% in Katavi to 100% in Kusini Unguja [9]. Despite relatively high national coverage, several regions failed to reach the recommended coverage threshold of 95% required for measles and rubella elimination, leaving pockets of susceptible populations [9].

Recent epidemiological evidence further underscores these challenges. In July 2022, a measles outbreak was confirmed in Tanzania, in accordance with the WHO Measles Outbreak Guidelines (2022), predominantly affecting children under five years and those aged 7–15 years [10]. The outbreak was initially detected in Dar es Salaam and subsequently spread to coastal regions, including Tanga and Pwani. Similar outbreaks were also reported in Zanzibar, with laboratory-confirmed cases identified in multiple districts across Unguja and Pemba [11]. These outbreaks highlight the continued vulnerability of certain regions and populations, even in the context of previous vaccination campaigns.

In response to these outbreaks and to sustain the gains achieved during the 2019 MR campaign, the Ministry of Health (MoH) in Tanzania, conducted a follow-up MR campaign in February 2024. The campaign targeted children aged 9–59 months, a critical age group for both measles and rubella vaccination. Evaluating the performance of this campaign is essential to identify residual coverage gaps and inform future immunization strategies, particularly in underserved and high-risk areas [2].

Spatial analysis offers a powerful and policy-relevant approach for revealing geographic inequities in vaccination coverage, enabling identification of priority areas for targeted, resource-efficient immunization interventions. Spatial distribution studies highlight that geographical barriers, socio-economic factors, marital status, higher poverty levels, parental education, and rural-urban divides, significantly impact of measles-rubella vaccine accessibility [12,13]. Addressing these barriers requires a multifaceted approach, including targeted health education and improved healthcare delivery systems and context-specific interventions, [12], particularly in high-risk areas identified through spatial analysis [13].

In assessing spatial patterns of measles-rubella campaign coverage survey, researchers typically examine population distribution, vaccination rates, and healthcare access to understand how local contexts shape coverage outcomes [14]. Spatial analytical techniques include disease mapping, spatial clustering, and risk factor identification through map comparisons [15]. Spatial clustering is particularly effective in pinpointing areas with low vaccination coverage, which are often at higher risk for outbreaks due to shared environmental or socio-economic factors influencing vaccine access and uptake [16]. Cuzick and Edwards (1990) identified three methods for detecting spatial clustering: cell count data, autocorrelation of neighboring cells, and analyzing the distance between vaccination events or coverage rates [17]. Advanced numerical methods like scan statistics [18], statistics by Ohno et al [19], Poisson statistics [20], Global Moran’s I [21], Global Geary’s C [22], General Getis-Ord’s G [23], and Local Moran’s I [24] are critical for detecting spatial clusters in health surveys. Hotspot analysis using Getis-Ord Gi* statistics (pronounced G-i-star) reveals clustering intensity, with positive scores indicating hotspots—areas with high coverage and negative scores showing cold spots—areas with low coverage [25].

Several studies have shown that mapping spatial distribution can effectively guide vaccination strategies, allowing public health officials to prioritize interventions in underserved areas and optimize resource allocation [15,26]. In Tanzanian context, as in other countries, utilizing spatial data to assess measles-rubella vaccine campaign coverage can play a crucial role in: i) identifying coverage gaps, ii) uncovering factors that drive disparities in vaccine coverage across various areas, iii) tackling spatial and demographic inequities in vaccine distribution, and iv) enabling more focused and efficient vaccination efforts [2,26,27]. Therefore, this study aimed to investigate the spatial distribution and factors influencing 2024 measles-rubella campaign coverage among children aged 9–59 months in mainland Tanzania.

## Materials and methods

### Study design and setting

A cross-sectional survey employing quantitative methods was conducted to assess coverage achieved during the 2024 measles–rubella campaign in Mainland Tanzania. The quantitative data were collected by interviewing caregivers of children 9–59 months within the selected households. A household was defined as a group of individuals who shared a common cooking pot or kitchen, consistent with national survey definitions. The primary target population for the post-campaign evaluation was children aged 9–59 months. The survey was conducted across all regions of Mainland Tanzania following completion of the MR campaign in February 2024, ensuring national representativeness.

### Sample size

The sample size was determined to ensure accurate, age- and sex-specific MR vaccine coverage estimates for children aged 9–59 months at the national level. With a target precision of ±5% and an expected coverage rate of 95%, a total of 14,040 households were required for the sample size.

### Sampling procedures

The study employed a two-stage stratified cluster sampling approach [28]. The first stage involved selecting Primary Sampling Units (PSUs) or enumeration areas (EAs) from Tanzania National Bureau of Statistics (NBS). PSUs were selected randomly using a probability proportional to size (PPS) method to ensure that larger population areas had a higher chance of selection, leading to more accurate and representative results. These PSUs represented the various regions of Mainland Tanzania, capturing a diverse demographic for the study. In the second stage, Secondary Sampling Units (SSUs) were selected within each EA. SSUs were households with at least one child aged 9–59 months, the target population for the measles-rubella campaign. In this study, each EA was listed, and a sketch map of the area was created to accurately locate the selected households. Households were assigned identification numbers for tracking.

To randomly select households, a randomizer tool was used to choose eight households per EA. If a household could not be located or did not meet the eligibility criteria, no replacement was made, and those households were excluded from the study. Additionally, households that did not provide consent or could not be accessed after three extended visits were excluded from the final sample. All eligible caregivers aged 18 or older in the selected households were interviewed. This included both regular residents and visitors who slept in the household the night before the survey. Interviewers used the household identification number and sketch map to locate and confirm the eligible households. The interviews focused on gathering data regarding vaccination status, demographic factors, and any barriers to vaccine access to understand the spatial patterns of vaccine coverage, factors contributing to coverage disparities, and the effectiveness of the 2024 measles-rubella campaign.

### Data collection

A total of 5 scientists from IHI, 26 regional supervisors, and 84 interviewers from all 26 regions in Mainland Tanzania attended training and a pre-test in Dar es Salaam. An average of 3 interviewers were selected for each region from the IHI database. The interviewers and regional supervisors were trained to understand the survey objectives, importance, and questionnaire. They were also trained in interview techniques to maximize response rates and ensure data validity. The roles of each study team member were clarified during the training. A training manual was prepared to enhance training effectiveness and serve as a reference during data collection. The study instruments were pilot-tested in an excluded EA to ensure understanding, correct wording of questions, and capture the intended data. Pilot testing also helped to estimate survey duration and confirm the survey teams’ understanding of sampling households in the field.

All quantitative data were collected by well-trained interviewers who have experience in administering quantitative survey studies. The data were collected using mobile devices (tablets) that were programmed with the Open Data Kit (ODK) application. All eligible respondents were interviewed at their households to determine if their children had received the MR vaccine during the campaign. The primary caregiver of children aged 9–59 months was administered a structured questionnaire to collect data for the evaluation of the MR vaccination campaign. These questionnaires assessed the vaccination status for MR, key demographic information, household socio-economic status (SES), demand-side constraints, and perceptions about the campaign.

The data collectors were required to check if the left-hand little fingers of the children were marked with special ink during the campaign as a certification of vaccination. If there was no mark, caregivers were asked to recall whether their children had received the MR vaccine by inquiring about the specific MR vaccination procedure. The MR vaccination procedure followed these steps: caregivers were asked if they remembered whether their children received the measles-rubella vaccine during the campaign. If yes, they were prompted to recall when and where the vaccination took place. Caregivers were also asked if they were informed or reminded about the vaccine during the campaign. Additionally, caregivers were inquired whether their child received the vaccine via injection in the left shoulder during the campaign period. Clinic card marks were also used as part of the assessment process.

### Data quality control

Quality control was vital for this assignment, with the Ministry of Health (MoH) staff collaborating closely with the senior study team during fieldwork. A skilled research team conducted the survey, using detailed field tools developed in partnership with MoH staff, which were piloted in training sessions. Each team had a field supervisor ensuring daily quality control, with supervisors accompanying at least one interview daily and randomly reviewing later interviews. Completed questionnaires were checked daily by interviewers and supervisors. The research team provided close oversight during training, data collection, entry, cleaning, and validation. Initial data cleaning was performed by interviewers on tablets before online uploads, with data clerks correcting errors using a user guide. A second data cleaning level occurred post-download by the Data manager, involving interviewers for clarification on discrepancies. Daily field reports from the regional data manager/supervisor further supported the quality control process.

### Primary outcome and explanatory variables

In this study the outcome variables were measles-rubella campaign coverage for children aged 9–59 months coded as “Yes” if their children had received the MR vaccine during the campaign and “No” if the child failed to receive the vaccine. Information on vaccination status during the campaign was obtained in the following ways; initially, finger markers were intended to be used to verify the coverage. The Post-MR campaign vaccination coverage survey was conducted in June, 2024 throughout mainland Tanzania, after the campaign implementation in February 2024, but verification of children’s vaccinations using finger markers was not possible due to significant delays in fieldwork. As a result, historical information or the caregiver’s recall was relied upon to assess coverage, supplemented by follow-up questions to confirm the caregivers’ awareness of the marked finger and the method of vaccine administration. The predictor’s variables were the caregiver’s age, marital status, sex of household head, sex of the child, caregiver’s education level, household size, occupation, wealth index, residence, and regions.

### Data management and data analysis

Data extraction, cleaning, weighting, and recording were done before any statistical analysis. During the data management process, MR vaccination campaign coverage was categorized into high coverage (≥85.2%) and low coverage (<77.1%) based on the distribution of regional coverage estimates observed in the study dataset and supported by both national and global epidemiological evidence. Previous post–MR campaign surveys conducted in Tanzania reported vaccination coverage levels of 89.0% in 2014, 88.25% in 2019, and 81.5% in 2024, demonstrating a progressive decline in coverage and the potential accumulation of susceptible populations over time [9,29,30]. Similarly, recent post-campaign evaluations in Tanzania have shown substantial regional disparities in MR coverage, ranging from 69% to 100%, despite a national average of approximately 88% [31]. The selected thresholds were further informed by the 2023 global mean coverage estimates for the first and second doses of measles-containing vaccine (MCV1: 85.2%; MCV2: 77.1%) reported by Pere Plans-Rubió etal in 2025 [32]. Descriptive statistics such as cross-tabulations and frequency tables were used to describe the characteristics of the study population

### Spatial and hotspots analysis

In this study, spatial autocorrelation was applied to assess vaccination coverage in the area. The Moran’s Index (Moran’s I) measured spatial patterns, determining if coverage was clustered, dispersed, or random [33], with significant values (p < 0.05) confirming spatial patterns [34].

The Moran’s I was calculated using the standard formula:


$$\begin{array}{c} 𝐌𝐨𝐫𝐚𝐧\text{'}𝐬\;𝐈=(𝐧/\Sigma \Sigma \;{𝐰}_{𝐢𝐣})\;\times \;[\Sigma \Sigma \;{𝐰}_{𝐢𝐣}\;({𝐱}_{𝐢}\;-\;\overset{―}{𝐱})({𝐱}_{𝐣}\;-\overset{―}{𝐱})]\;/\;[\Sigma \;({𝐱}_{𝐢}\;-\overset{―}{𝐱}{)}^{2}] \end{array}$$

where:

n = number of spatial units (regions)
xᵢ, xⱼ = MR vaccination coverage in regions i and j
x̄ = mean MR vaccination coverage
wᵢⱼ = spatial weight representing the neighborhood structure

The autocorrelation analysis was conducted at regional levels to capture localized variations and broader spatial patterns. A total of 26 regions and 1,483 sub – regions represented as enumeration areas in this study were analysed using first-order queen contiguity to define spatial neighbours, considering all regions sharing a common boundary. A row-standardized spatial weight matrix was applied consistently across Moran’s I analyses and Getis-Ord Gi* analyses to account for spatial dependence among neighbouring units. Following global Moran’s I estimation, Local Indicators of Spatial Association (LISA) were calculated to identify local clusters and outliers, including high-high, low-low, high-low, and low-high regions, which are critical for guiding targeted health interventions [33,35]. Yanguang Chen in 2024 provides a refined theoretical formulation of LISA, emphasizing the need for normalized and mathematically consistent local statistics that properly align with global measures of spatial autocorrelation, thereby improving the interpretation of spatial clusters and outliers in geographic data [36].

Hotspot analysis using the Getis-Ord Gi* statistic was applied in this study to identified areas of high and low measles-rubella vaccination coverage. The Getis–Ord Gi* was calculated using the standard formula:

$𝐆𝐞𝐭𝐢𝐬-𝐎𝐫𝐝\;{𝐆𝐢}^{*}=[\Sigma \;{𝐰}_{𝐢𝐣}\;{𝐱}_{𝐣}\;-\;\overset{―}{𝐗}\;\Sigma \;{𝐰}_{𝐢𝐣}]\;/\;[𝐒\;\times \;𝐬𝐪𝐫𝐭((𝐧\;\Sigma \;{𝐰}_{𝐢𝐣}^{2}\;-\;(\Sigma \;{𝐰}_{𝐢𝐣}{)}^{2}\left(𝐧-1))\right]$

where:

xⱼ = value of the variable at location j (e.g., vaccination coverage)
wᵢⱼ = spatial weight between locations i and j (based on contiguity or distance)
n = total number of spatial units
X̄ = mean of the variable across all units
S = standard deviation of the variable across all units

Getis–Ord Gi* statistics were used to identify spatial clustering of MR campaign coverage. The Getis-Ord Gi* statistics use the z-scores, the positive z-scores indicated the hotspots—areas with high MR campaign coverage, while the negative z-scores highlighted the cold spots—areas with low MR campaign coverage. Confidence limits for Gi* statistics were calculated at the 95% confidence level (±1.96) to ensure consistency with standard statistical reporting practices in epidemiological studies. Areas with z-scores ≥1.96 were classified as hotspots (high-coverage areas surrounded by high-coverage neighbors), while areas with z-scores ≤−1.96 were classified as cold spots (low-coverage areas surrounded by low-coverage neighbors) at the 95% significance level. Areas not meeting this threshold were classified as not significant.

The spatial clustering results were visualized using standard LISA cartographic conventions where High- High clusters (enumeration areas with high coverage surrounded by high-coverage neighbors, i.e., hot spots) were presented with green shades. The red shades represent Low-Low clusters (enumeration areas with low MR coverage surrounded by low-coverage neighbors, i.e., cold spots). The white shades indicate spatial outliers which are High-Low or Low-High enumeration areas respectively. Also, the white shades indicate non-significant clustering. Interpretation of spatial clustering followed standard spatial epidemiology definitions of hotspot (High-High clusters), cold spot (Low-Low clusters), and spatial outliers (High-Low and Low-High outliers) [23,24]. In all maps from this study the high coverage (favorable outcome) was given a green colour and unfavorable outcome was given a red colour.

In this study, inferential spatial regression models were not fitted because adequate district- and ward-level covariate data were not available. Instead, spatial autocorrelation analyses were conducted at both regional and sub-regional administrative levels to examine spatial clustering in vaccination coverage and to explore potential scale-related effects, including those related to the Modifiable Areal Unit Problem (MAUP) [37–39]. Using these administratively meaningful units enabled robust identification of micro-level hotspots and cold spots, generating reproducible spatial patterns to guide targeted interventions, resource allocation, and coordinated vaccination planning.

### Logistic regression analysis

To identify factors associated with the campaign coverage, we used a multivariable multi-level logistic regression model. After fitting a bi-variable logistic regression analysis, variables with a *p-value* of *<* 0.2 in the bi-variable analysis were further considered in the multivariable multi-level logistic regression model. *Adjusted odds ratio* (AOR) with 95% *CI* and *p-value <*0.05 in the multivariable multi-level logistic regression model were used to declare statistically significant variables associated with campaign coverage. In this study, we use the R software [40] to execute the data analysis.

#### Software.

All analyses were conducted using R statistical software (version 4.3.1) [40]. Spatial autocorrelation were performed using the *spdep*. Spatial data handling and mapping were conducted with *sf* and *tmap*, and hotspot analyses were implemented using *spatstat*.

### Ethics statement

This study was conducted by an experienced research team, ensuring that national, regional, and district health authorities were provided with sufficient information about the study. Written informed consent was obtained from the head of each household and translated into Swahili (Tanzania national language) to explain the purpose, the researchers involved, and the study’s details. The translated consent was also used to introduce the survey team to community leaders. The study followed the International Ethical Guidelines for Biomedical Research Involving Human Subjects [41], as recommended by the WHO. Verbal informed consent was used for a standard coverage survey like this, as it was non-intrusive and did not involve sensitive information. A standard explanation and introduction were provided to each caregiver before seeking individual informed consent through a proxy. Caregivers were allowed to ask questions and indicate their willingness to participate in the survey. The confidentiality of all participants was ensured, and the surveys posed no risk to them. Any participant with an acute illness was referred to a healthcare facility. Ethical approval was obtained from the Ethical Committee of Ifakara Health Institute (IHI/IRB/No: 14–2024).

## Results

A total of 16703 caregivers with children aged 6–59 months participated in this study. Thirteen thousand six hundred and two (81.5%) of the children received measles-rubella vaccination during the campaign. There is an equal proportion in terms of sex of children who received measles-rubella vaccination during the campaign. The majority of children’s received the measles-rubella vaccine lived with caregiver’s who attained primary education level. Eight thousand nine hundred and thirty-five (65.7%) of the vaccinated children during the campaign lived in rural areas. The majority of the surveyed caregivers were married or cohabiting. Regarding wealth, 5621 (41.3%) of vaccinated children during the campaign came from households with middle-level wealth (Table 1).

**Table 1:** Characteristics of study participants and MR coverage by various background characteristics among children aged 9–59 months in Tanzania.

Variables		Received MR		p-value^2^
		No	Yes	
	Overall, N = 16703^1^	n = 3101^1^	n = 13602^1^	
	N (%)	n (%)	n (%)	
Child sex				0.12
Male	8344 (50.0)	1588 (51.2)	6756 (49.7)	
Female	8359 (50.0)	1513 (48.8)	6846 (50.3)	
Caregiver’s age				<0.001
18–24	2819 (16.9)	603 (19.4)	2216 (16.3)	
25–34	7864 (47.1)	1488 (48.0)	6376 (46.9)	
34+	6020 (36.0)	1010 (32.6)	5010 (36.8)	
Caregiver’s education				
No formal education	2256 (13.5)	563 (18.2)	1693 (12.4)	
Primary education	10942 (65.5)	1937 (62.5)	9005 (66.2)	
Secondary education and more	3505 (21.0)	601 (19.3)	2904 (21.4)	
Residence				<0.001
Rural	10858 (65.0)	1923 (62.0)	8935 (65.7)	
Urban	5845 (35.0)	1178 (38.0)	4667 (34.3)	
Caregiver’s marital status				0.46
Married/Cohabited	14093 (84.4)	2603 (83.9)	11490 (84.5)	
Others	2610 (15.6)	498 (16.1)	2112 (15.5)	
Wealth status				<0.001
Poor	3600 (21.6)	840 (27.1)	2760 (20.3)	
Middle	6774 (40.5)	1153 (37.2)	5621 (41.3)	
Rich	6329 (37.9)	1108 (35.7)	5221 (38.4)	

^1^n (%), ^2^Pearson’s Chi-squared test.

### Measles-Rubella vaccination campaign coverage in Mainland Tanzania

**Fig 1:**
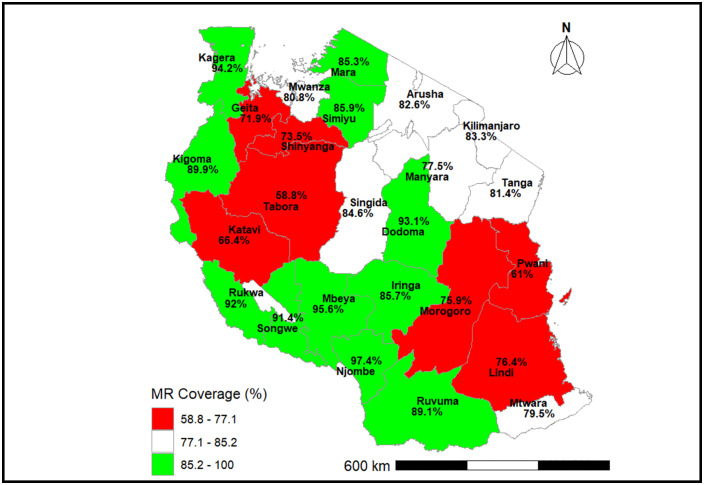
Map of MR vaccination rate among children aged 9–59 months across regions of Tanzania, MR survey 2024. Map generated by the authors using R (R Core Team, 2026) with Tanzania administrative boundary shapefiles and geospatial survey data provided by the Ifakara Health Institute. Publication of this figure was authorized by the IHI Institutional Review Board under the Creative Commons Attribution License (CC BY 4.0).

In mainland Tanzania, an estimated 81.5% (95% CI: 80.0%−82.9%) of the children aged 9–59 months received the MR campaign dose, as assessed by caregivers’ recall. The coverage varied among different regions, with Tabora and Pwani having a low coverage rate of 58.8% (95% CI: 51.3%−65.9%) and 61.0% (95% CI: 45.0%−75.0%) respectively. The Njombe and Mbeya demonstrated a high coverage rate of 97.4% (95% CI: 90.5%−99.3%) and 95.6% (95% CI: 90.9%−97.9%) respectively. The estimates of the measles-rubella campaign coverage are presented in Table 2 and Fig 1.

**Table 2:** Estimated measles-rubella campaign vaccination coverage – caregiver’s recall.

Region	Weighted %	95%CI	n	N
Njombe	97.4	90.5-99.3	167	172
Mbeya	95.6	90.9-97.9	570	601
Kagera	94.2	90.5-96.5	1,005	1072
Dodoma	93.1	90.5-95.0	709	777
Rukwa	92.0	85.6-95.6	329	363
Songwe	91.4	80.4-96.5	286	307
Kigoma	89.9	83.6-94.0	763	864
Ruvuma	89.1	82.8-93.3	267	298
Simiyu	85.9	80.4-90.0	1,032	1213
Iringa	85.7	76.4-91.7	143	168
Mara	85.3	80.4-89.2	893	1033
Singida	84.6	76.5-90.3	375	460
Kilimanjaro	83.3	75.7-88.9	401	478
Arusha	82.6	75.7-87.9	394	462
Tanga	81.4	74.2-87.0	422	513
Mwanza	80.8	75.2-85.5	1,093	1346
Mtwara	79.5	72.5-85.1	298	365
Dar Es Salaam	78.4	74.3-82.1	889	1163
Manyara	77.5	68.9-84.3	435	564
Lindi	76.4	52.5-90.5	196	232
Morogoro	75.9	67.2-82.9	507	647
Shinyanga	73.5	63.8-81.3	444	643
Geita	71.9	65.2-77.8	814	1168
Katavi	66.4	48.8-80.4	249	343
Pwani	61.0	45.0-75.0	188	272
Tabora	58.8	51.3-65.9	733	1179
**Overall**	**81.5**	**80.0-82.9**	**13,602**	**16703**

### Spatial autocorrelation and hotspot analysis of MR campaign coverage

The spatial distribution of MR coverage in Mainland Tanzania was found to be non-random (*Moran’s I* = 0.34, *p-value* < 0.01). The Moran’s Index value observed (0.34) exceeded the expected value (−0.004), and the p-value was statistically significant (<0.01).

### The Local Indicators of Spatial Association (LISA)

**Fig 2:**
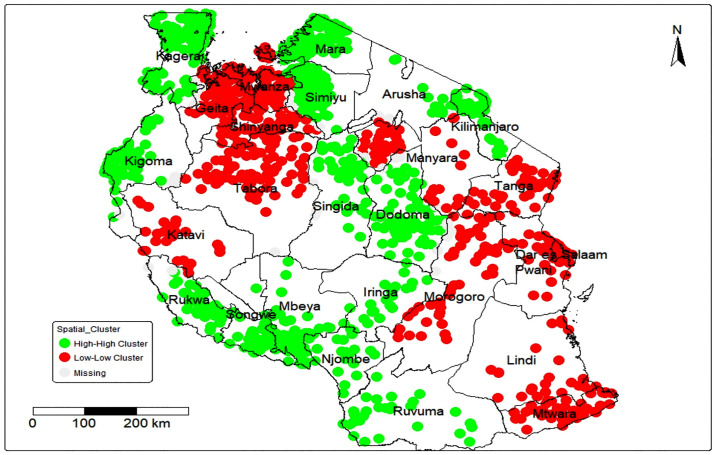
Local Indicators of Spatial Association (LISA) cluster map of MR vaccination coverage among children aged 9–59 months across Tanzania, MR survey 2024. *Green shades represent High–High clusters (enumeration areas with high MR coverage surrounded by high-coverage neighbours, i.e., hot spots), while red shades represent Low–Low clusters (enumeration areas with low MR coverage surrounded by low-coverage neighbours, i.e., cold spots). White shades indicate spatial outliers corresponding to High–Low or Low–High enumeration areas, respectively. Also, white shades indicate non-significant clustering. Map generated by the authors using R (R Cor*e Team, 2026) wit*h Tanzania administrative boundary shapefiles and geospatial survey data provided by the Ifakara Health Institute. Publication of this figure was authorized by the IHI Institutional Review Board under the Creative Commons Attribution License (CC BY 4.0).*

The LISA cluster map on Fig 2 identified statistically significant High–High and Low–Low clusters of MR vaccination coverage, as well as spatial outliers. Low–Low clusters (cold spots), defined as areas with low vaccination coverage surrounded by areas with similarly low coverage, were concentrated in Tabora, Katavi, and parts of Pwani and Dar es Salaam. High–High clusters (hotspots), defined as areas with high vaccination coverage surrounded by high-coverage neighbors, were observed in Njombe, Mbeya, Kagera, and Rukwa.

### Getis–Ord Gi* hotspot analysis

**Fig 3:**
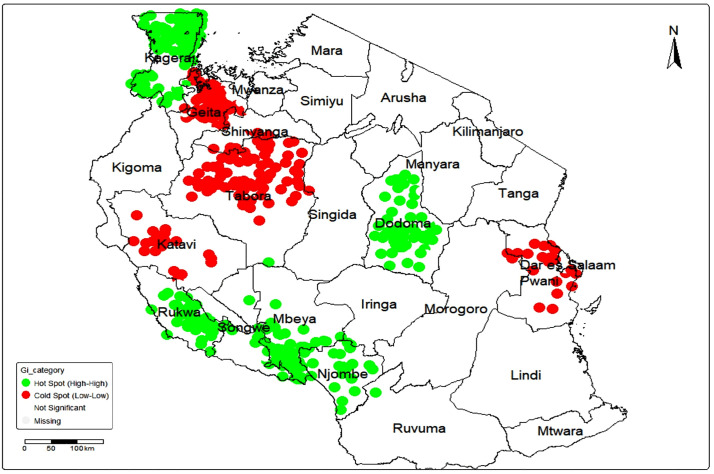
Spatial distribution of statistically significant hotspots and cold spots of MR coverage across Tanzania, MR survey 2024 using Getis–Ord Gi* analysis. *Green shades represent hotspots (high values surrounded by high values), red shades represent cold spots (low values surrounded by low values), at 95% confidence level,* white *shades indicate non-significant clustering or missing data. Map generated by the authors using R (R Core Team, 2026) with Tanzania administrative boundary shapefiles and geospatial survey data provided by the Ifakara Health Institute. Publication of this figure was authorized by the IHI Institutional Review Board under the Creative Commons Attribution License (CC BY 4.0).*

Fig 3 presents the results of the Getis–Ord Gi* local spatial autocorrelation analysis, illustrating statistically significant spatial clustering of the MR coverage across Tanzania at the regional level. The analysis identifies hotspots (areas with significantly high values surrounded by high values) and cold spots (areas with significantly low values surrounded by low values) at the 95% confidence level, while areas shown in white indicate non-significant clustering or missing data.

Statistically significant hotspots at 95%CI, shown in green, are predominantly concentrated in the southern highlands, particularly in Njombe and parts of Mbeya regions, indicating a strong spatial concentration of high MR coverage. Additional hotspots at 95% confidence level) extend into neighbouring regions such as Songwe and Rukwa, suggesting spatial spillover effects and regional clustering.

In contrast, statistically significant cold spots at 95% CI, represented in red, are observed mainly in western Tanzania, notably in Tabora and Katavi regions, as well as in parts of the eastern coastal zone, including Dar es Salaam and Pwani. Cold spots at 95% CI extend into Shinyanga, Geita, and Mwanza, reinforcing the presence of a broader low-value cluster in the north-western corridor.

### Multi-level mixed-effects logistic regression analysis

The multi-level mixed-effects logistic regression analysis identified several significant individuals- and community-level determinants of measles–rubella (MR) vaccination coverage during the campaign among children aged 9–59 months in Tanzania, after adjusting for clustering at the enumeration area level.

#### Individual-level factors.

Child sex was not significantly associated with MR vaccination coverage during the campaign, although male children had slightly lower odds of receiving MR vaccination compared to females (AOR = 0.92, 95% CI: 0.84–1.01, p = 0.065). Children of younger mothers were less likely to be vaccinated compared to those whose mothers were aged ≥35 years. Specifically, maternal age 18–24 years (AOR = 0.72, 95% CI: 0.62–0.82, p < 0.001) and 25–34 years (AOR = 0.80, 95% CI: 0.72–0.89, p < 0.001) were both associated with reduced odds of receiving MR vaccination. Children of married/cohabiting mothers had comparable odds of receiving MR vaccination relative to those in other marital categories (AOR = 1.04; 95% CI: 0.92–1.19; p = 0.499).

Maternal education was an important predictor of receiving MR vaccination during the campaign. Compared to caregiver’s with secondary education or higher, children of caregivers with no formal education had significantly lower odds of receiving MR vaccination (AOR = 0.72, 95% CI: 0.60–0.86, p < 0.001). Primary education showed a borderline association of receiving MR vaccination (AOR = 0.89, 95% CI: 0.78–1.01, p = 0.063), while caregiver’s who reported “don’t know” educational status had substantially lower odds of receiving MR vaccination (AOR = 0.41, 95% CI: 0.17–1.00, p = 0.049).

Household wealth status was also significantly associated with receiving MR vaccination during the campaign. Children from poor households had lower odds of receiving MR vaccination compared to those from middle-income households (AOR = 0.69, 95% CI: 0.60–0.79, p < 0.001). In contrast, children from rich households had slightly higher odds of receiving MR vaccination (AOR = 1.16, 95% CI: 1.01–1.33, p = 0.036).

#### Community-level factors.

Residence was significantly associated with receiving MR vaccination during the campaign. Children residing in urban areas had lower odds of receiving MR vaccination compared to those in rural areas (AOR = 0.72, 95% CI: 0.60–0.87, p < 0.001).

Regional differences were pronounced, with substantial geographic heterogeneity in MR vaccination coverage. Compared to Dar es Salaam being the capital city, significantly higher odds of receiving MR vaccination during the campaign were observed in several regions, including Njombe (AOR = 15.90, 95% CI: 4.54–55.67, p < 0.001), Mbeya (AOR = 9.22, 95% CI: 4.82–17.65, p < 0.001), Kagera (AOR = 7.65, 95% CI: 4.50–13.01, p < 0.001). Conversely, lower odds of receiving MR vaccination during the campaign were observed in Tabora (AOR = 0.46, 95% CI: 0.30–0.71, p < 0.001). Several other regions, including Songwe, Rukwa, Kigoma, Dodoma, Arusha, Mara, Singida, Tanga, Mwanza, and Ruvuma, also showed significantly higher odds of vaccination, whereas some regions such as Geita, Katavi, Kilimanjaro, Morogoro, Mtwara, Shinyanga, and Pwani did not show statistically significant differences (Table 3).

**Table 3:** Multi-level mixed-effects logistic regression analysis of individual and community-level factors associated with measles vaccination among children aged 9–59 months in Tanzania.

Variables	AOR	95% CI	p-value
**Individual-level factors**			
**Child sex**			
Female	Ref	–	–
Male	0.92	0.84–1.01	0.065
**Maternal age category**			
≥35 years	Ref	–	–
18–24 years	0.72	0.62–0.82	<0.001
25–34 years	0.80	0.72–0.89	<0.001
**Marital status**			
Others	Ref	–	–
Married/Cohabiting	1.04	0.92–1.19	0.499
**Maternal education**			
Secondary education and above	Ref	–	–
No formal education	0.72	0.60–0.86	<0.001
Primary education	0.89	0.78–1.01	0.063
Don’t know	0.41	0.17–1.00	0.049
**Household wealth status**			
Middle	Ref	–	–
Poor	0.69	0.60–0.79	<0.001
Rich	1.16	1.01–1.33	0.036
**Community-level factors**			
**Residence**			
Rural	Ref	–	–
Urban	0.72	0.60–0.87	<0.001
**Region**			
Dar es Salaam	Ref	–	–
Arusha	2.05	1.15–3.66	0.015
Dodoma	4.39	2.58–7.46	<0.001
Geita	0.70	0.44–1.11	0.133
Iringa	2.04	0.92–4.53	0.081
Kagera	7.65	4.50–13.01	<0.001
Katavi	0.76	0.42–1.38	0.370
Kigoma	3.82	2.28–6.41	<0.001
Kilimanjaro	1.56	0.89–2.75	0.124
Lindi	2.01	0.95–4.24	0.068
Manyara	1.15	0.67–2.00	0.607
Mara	2.42	1.50–3.90	<0.001
Mbeya	9.22	4.82–17.65	<0.001
Morogoro	1.16	0.72–1.87	0.534
Mtwara	1.41	0.78–2.57	0.255
Mwanza	1.56	1.00–2.42	0.049
Njombe	15.90	4.54–55.67	<0.001
Pwani	0.53	0.28–1.02	0.058
Rukwa	4.10	2.10–7.98	<0.001
Ruvuma	2.94	1.46–5.92	0.003
Shinyanga	0.76	0.46–1.24	0.271
Simiyu	2.42	1.51–3.87	<0.001
Singida	1.81	1.02–3.23	0.044
Songwe	6.22	2.95–13.11	<0.001
Tabora	0.46	0.30–0.71	<0.001
Tanga	1.72	1.01–2.94	0.048

OR = Odds Ratio; AOR = Adjusted Odds Ratio; CI = Confidence Interval; Ref = Reference category.

## Discussion

This study revealed that overall 81.5% of children aged 9–59 months in Mainland Tanzania had received the measles-rubella vaccine during the campaign which is lower than the previous coverage of the MR vaccination in 2019 which was 88.2% [9]. The coverage of the 2024 MR vaccine did not meet the global target of 95% or higher [42] of the population must be vaccinated [43,44], means many targeted children were not reached during the MR vaccination campaign in Mainland Tanzania. However, we demonstrated that the overall measles-rubella coverage among children aged 9–59 months in Mainland Tanzania was low as global target [42], which is similar to other low- and middle-income countries (LMICs) such as Ethiopia (58.8%), but lower than Afghanistan (66%) and Pakistan (76%) [45]. A possible explanation on this disparity in vaccination coverage is likely influenced by a range of factors, both distinct and cross-cutting. Notably, barriers such as limited healthcare access in remote areas, vaccine supply interruptions, insufficient public awareness, cultural opposition, and inadequate outreach to marginalized communities contribute significantly. Cross-cutting issues, including widespread vaccine mistrust driven by misinformation, economic constraints like transportation costs, and ineffective communication strategies, further exacerbate vaccine hesitancy.

In this study, our findings show that the measles-rubella vaccination coverage was highest in Njombe region which was 97.4%, and lowest in the Tabora region which was 58.8%. This result is supported by previous studies in Tanzania that showed childhood vaccination coverage varied across different regions of the country [9,46]. The 2022 Tanzania Demographic and Health Survey and Malaria Indicator Survey reported a national decline in full vaccination against all basic antigens coverage for children aged 12–23 months, from 75% in the years 2015–16 to 53%, with regional coverage ranging from 3% in Shinyanga to 66% in Kilimanjaro [47]. The disparity between Njombe and Tabora underscores the need for focused interventions and resource allocation. Improving vaccination coverage in underperforming regions can reduce measles and rubella burdens, contributing to better public health and advancing global vaccination goals.

In this study, the global Moran’s I was estimated at 0.34 (p < 0.05), indicating a moderate positive spatial autocorrelation. The observed Moran’s I value of 0.34 indicates moderate positive spatial autocorrelation and is comparable to values reported in similar immunization studies in sub-Saharan Africa (range: 0.30–0.42), reinforcing the presence of geographically structured vaccination inequities [33]. These cold- spots of under-vaccination are spatially aggregated, suggesting that the risk of measles transmission is not isolated but follows a broader regional geographic pattern, consistent with spatial dependence in immunization coverage. Such areas are priority zones for catch-up vaccination campaigns, as they represent vulnerable transmission pockets where population immunity is likely below the 95% threshold required to interrupt measles transmission [48].

In contrast, clusters of high measles-rubella (MR) coverage indicate areas where the Expanded Programme on Immunization (EPI) has achieved geographic synergy, potentially reflecting stronger health system performance, higher maternal education, and urban advantages. Studies in Tanzania, Malawi, and other East African countries have consistently shown that maternal education, urban residence, and proximity to health facilities are strongly associated with higher vaccination uptake [49]. However, the presence of spatial outliers suggests that localized ward-level factors—such as health facility performance, staffing, or intermittent vaccine stock-outs—may override broader regional trends. Evidence from Ethiopia [50], Uganda [51], and the Democratic Republic of Congo [52] indicates that vaccine stock-outs, logistical challenges, and variable health service quality can lead to pockets of low immunization coverage, even within regions that are otherwise performing well. Such micro-level inequities highlight the need for targeted interventions at the ward or facility level to ensure equitable vaccination coverage and to strengthen the resilience of immunization programs against local disruptions [53].

This study also demonstrates pronounced spatial heterogeneity in the distribution of the MR coverage across Tanzania, with statistically significant hotspots and cold spots identified using the Getis–Ord Gi* statistic. The presence of hotspots in the southern highlands, particularly in Njombe and Mbeya regions, suggests localized clustering that is unlikely to be due to random variation. Similar spatial clustering patterns have been widely reported in public health and infectious disease studies, where geographic context plays a critical role in shaping population-level outcomes [23,54]. The identification of persistent cold spots in western regions such as Tabora and Katavi, as well as in the eastern coastal zone including Dar es Salaam and Pwani, highlights important geographic inequities. In infectious disease epidemiology, clusters of persistently low program performance or health outcomes can undermine national-level gains by sustaining transmission and increasing outbreak risk [35,54,55]. Urban coastal settings, in particular, are often characterized by high population mobility and informal settlements, which may contribute to weaker program reach and uneven service delivery [56].

The absence of statistically significant clustering in several central and southern regions may reflect heterogeneous local conditions or insufficient spatial dependence to detect clustering at the selected scale. This phenomenon is consistent with previous studies emphasizing that national or regional averages can mask important sub-national variation, reinforcing the need for spatially explicit analyses in health monitoring [23,54]. From a methodological perspective, the Getis–Ord Gi* approach provides a robust framework for identifying localized clusters and supports precision public health interventions, allowing resources to be targeted to areas of greatest need [23]. Integrating such spatial analyses into routine surveillance systems can improve the equity and effectiveness of infectious disease control strategies, particularly in low-resource settings where efficient allocation of limited resources is critical [56,57]. Overall, these findings underscore the importance of incorporating spatial epidemiological methods into program evaluation and policy planning. By identifying geographic pockets of vulnerability, policymakers and program managers can design targeted interventions that address structural and contextual barriers, thereby strengthening population-level disease prevention and control.

The potential influence of the modifiable areal unit problem (MAUP) was explicitly considered, as spatial patterns of MR vaccination coverage can vary depending on the spatial scale and zoning of administrative units used in the analysis. MAUP has been documented to impact spatial clustering results in health and environmental research by altering the strength and location of identified clusters when different spatial units are used [58]. In our analysis, clustering patterns at the regional level were broadly consistent with ward level results, but we acknowledge that ward boundary definitions can influence local cluster identification.

Spatial dependence in MR coverage was assessed using Moran’s I, LISA, and Getis Ord Gi* statistics. These exploratory spatial autocorrelation methods have been widely applied in vaccination and public health studies to detect statistically significant clustering [58,59]. Although spatial regression models—such as spatial lag or spatial error models—can be used to statistically model spatial dependence in predictors, they were not applied in this study [60]. The focus here was descriptive spatial mapping to identify hotspots and cold spots of MR coverage for public health planning, and ward level covariate data necessary for robust spatial regression were limited in completeness and availability. Moran’s I, LISA, and Gi* provided robust measures of spatial dependence appropriate for exploratory spatial characterization without strong modeling assumptions [35]. While LISA statistics are useful for identifying local spatial clustering, their interpretation should be considered exploratory rather than confirmatory. As noted in recent spatial epidemiology literature, LISA clusters may be sensitive to spatial scale and neighborhood definitions. Therefore, findings should be interpreted as indicators of geographic patterns requiring further investigation rather than definitive causal relationships.

Donors and policymakers are increasingly interested in mapping the spatial heterogeneity of childhood vaccination [61] to identify existing gaps and intervene accordingly. The spatial autocorrelation analysis of our data showed a clustering pattern of childhood measles vaccination coverage were mostly western, eastern, lake zone part of Tanzania. Studies in various countries, including Ethiopia [62], Mozambique [63], and Brazil [58], reveal significant spatial heterogeneity in vaccination coverage. For example, Ethiopia’s research showed clustering in regions like Afar and Amhara, while Mozambique identified regional disparities with lower coverage. In São Paulo, Brazil, varied measles vaccination rates were observed across municipalities. Contrastingly, the Somali Region in Ethiopia had lower coverage in zones like Nogob and Erer [64]. Factors like healthcare infrastructure, cultural practices, and data collection could explain these discrepancies. Tanzania needs further research to understand these clustering patterns, and collaboration with neighboring countries could inform targeted interventions. In addition; to address these disparities, targeted interventions must be tailored to the specific geographical, economic, and social conditions of each region.

In the multi-level mixed-effects logistic regression analysis, caregiver’s age, caregiver’s education, household wealth status, region, and place of residence were significantly associated with measles-rubella vaccination coverage.

### Individual-level factors

In this study, male children had slightly lower odds of the outcome compared to female children (AOR = 0.92, 95% CI: 0.84–1.01), although the association was not statistically significant at the 5% level (p = 0.065). The present finding that child sex was not a significant predictor of the outcome is consistent with recent high-level evidence from Sub-Saharan Africa. A 2026 systematic review and meta-analysis involving over 290,000 children reported that sex of the child is not a consistent determinant of full immunization after adjustment for maternal and household characteristics, with socioeconomic factors showing stronger predictive value [65]. Similarly, a 2025 critical review on gender-related barriers to immunization in Sub-Saharan Africa found that observed sex differences are largely context-specific and mediated by cultural and structural determinants rather than biological sex differences [66]. Although some studies in LMICs report female disadvantage in access to health services, more recent syntheses suggest that such disparities are not uniform across Sub-Saharan Africa and tend to diminish once household wealth, maternal education, and healthcare access are considered [67,68]. The possible explanation for this could be the observed borderline effect may reflect residual confounding or contextual variation rather than a true gender disparity. Overall, these results reinforce the predominance of household and maternal characteristics over biological sex in shaping child health service utilization.

Children lived with caregiver’s who had no formal education or those who do not know their education status had significantly lower odds of receiving MR vaccination as compared to caregiver’s with secondary education or higher. This is supported by previous studies done in Ethiopia [69], the Democratic Republic of Congo [70], and China [71]. A possible explanation might be that caregiver’s maternal education is vital to enhance awareness about childhood vaccination and to improve the utilization of primary health-care services, such as childhood vaccination services. Educated caregiver’s tend to have improved communication skills, which makes interactions with health-care providers easier, and leads to a better comprehension of vaccination programs and practices.

Maternal age was a pivotal factor in vaccination uptake, with children of younger mothers aged 18–24 years exhibiting significantly lower odds of MR vaccine coverage compared to those born to mothers aged 35 or older. This trend aligns with a broad body of global literature showing that older mothers generally possess greater maternal experience, socioeconomic autonomy, and health-seeking awareness, which translates directly into higher utilization of child health services [72,73]. Conversely, younger mothers frequently face systemic barriers, including restricted decision-making power, limited access to health information, and lower socioeconomic positioning, all of which compromise timely healthcare delivery for their children [74,75]. While some global evidence suggests that these age-based disparities diminish once underlying household wealth and education are accounted for, the patterns observed in Tanzania underscore a persistent vulnerability among young caregivers that warrants targeted programmatic communication and support.

The household wealth status was also significantly associated with receiving MR vaccination during the campaign. Children from poor households had lower odds of receiving MR vaccination compared to those from middle-income and rich households. Across the world, one common theme emerges in the fight against measles and rubella: socio-economic status plays a crucial role in vaccination coverage. In Vietnam [76], children from lower-income families, living in rural areas, and with less-educated mothers, were less likely to receive timely immunizations. Similarly, in Japan [77], higher income inequality led to lower vaccination rates, while communities with stronger social ties saw better coverage. Tanzania [9], Niger [78], Canada [79], and India [80] all echoed this pattern, with wealthier families more likely to vaccinate their children. The World Health Organization (2023) confirms that inequities in immunization, driven by socio-economic factors, continue to hinder global efforts to eliminate these preventable diseases [81]. The story is clear: addressing these disparities is key to ensuring every child, regardless of background, is protected from measles and rubella.

### Community-level factors

Place of residence was significantly associated with measles-rubella vaccination coverage in this study, with urban areas generally exhibiting higher vaccination rates than rural counterparts. This disparity is attributed to factors such as improved healthcare infrastructure, better access to vaccination services, and increased health awareness in urban settings. Our results revealed that, children in urban setting had lower chance of receiving the MR vaccination during the campaign compared to those children who lived in rural areas. This finding contrasts with evidence from Ethiopia, where urban residence was associated with higher vaccination coverage, highlighting the context-specific nature of urban–rural immunization dynamics [82]. Conversely, research in Pakistan which revealed that children residing in remote rural areas had higher measles vaccination coverage compared to urban areas, with rates of 89.8% in rural areas versus 75.2% in urban areas [83]. These contrasting findings underscore the complex relationship between place of residence and vaccination coverage, suggesting that factors such as healthcare accessibility, cultural practices, and local health policies play significant roles in vaccination rates.

This study reveals pronounced spatial heterogeneity in vaccine uptake across Tanzania, with regions like Njombe showing significantly higher odds of coverage compared to Dar es Salaam, while Tabora lags behind. These marked subnational variations mirror broader Tanzanian literature showing that childhood immunization is heavily dictated by localized health facility density, outreach intensity, and supply chain performance [7]. The elevated odds of coverage observed in the southern highlands likely reflect highly successful, campaign-driven interventions, whereas depressed odds in other regions point to hard-to-reach populations and critical subnational service delivery gaps. Ultimately, these findings emphasize that regional context is a vital determinant of child health outcomes, reinforcing the urgent need for targeted, place-based programmatic interventions to eliminate spatial inequities in Mainland Tanzania [8].

### Strength and limitation of the study

This study has its strengths and limitations. This study was strong in a number of ways. First, the researches team collected data from a variety of sources, such as vaccination cards, medical records, and mother/caregiver recall, were used to determine childhood vaccination coverage. The collection of high-quality data was also facilitated by the utilization of digital tools and the employment of skilled data collectors. Second, the analysis utilized a large nationally representative sample, enhancing the generalizability of the findings across Mainland Tanzania. Third, the application of multilevel mixed-effects logistic regression appropriately accounted for clustering at the community level and enabled simultaneous assessment of individual- and contextual-level determinants of vaccination uptake. In addition, the significant cluster-level variance further justified the use of multilevel analysis. Furthermore, the model identified substantial regional inequalities in measles-rubella vaccination coverage, highlighting regions with both high and low vaccination performance, which provides valuable evidence for targeted public health interventions and equitable immunization strategies. Finally, the overall model was statistically significant, indicating strong explanatory power for variations in vaccination uptake.

However, the study also had several limitations should be considered. First, the cross-sectional design limits causal inference between explanatory variables and vaccination uptake. In addition, including vulnerability to biases that are closely related to the cross-sectional study design, such as recall bias and non-response bias. Mothers or caregivers who lacked vaccination cards may have forgotten their child’s vaccination status, which could have led to misclassification. The survey was conducted 4–5 months after the MR vaccination campaign. The ink previously used to mark the fingers had already been removed, so the research team relied on the caregivers’ memory of events before and during the vaccine administration. However, this reliance on memory may have introduced some recall bias. To reduce this bias, research assistants reviewed asked the series of the questions related to the MR vaccine provision. Second, some potentially important factors, including healthcare accessibility, vaccine stock-outs, community vaccine hesitancy, media exposure, and cultural beliefs, were not available in the dataset and may have contributed to residual confounding. Finally, some regional estimates showed wide confidence intervals, suggesting limited precision in certain subgroup analyses.

## Conclusion

This study reveals pronounced spatial heterogeneity in measles–rubella vaccination coverage across Mainland Tanzania, with high-coverage clusters concentrated in the southern highlands and persistent low-coverage clusters in the western and eastern regions, alongside key socio-demographic determinants of vaccine uptake. These findings suggest that integrating geospatial insights with equity-focused planning may support more targeted public health interventions. Employing such spatially explicit, data-driven approaches not only provides actionable guidance for national immunization planning in Tanzania but also establishes a replicable framework for other low- and middle-income countries striving to close persistent vaccination gaps and achieve universal, equitable coverage.

## Supporting information

S1 Dataset Minimal dataset used for the analysis of spatial distribution and associated factors influencing 2024 measles-rubella vaccination campaign coverage among children aged 9–59 months in Mainland Tanzania. The dataset is provided in Stata (.dta) format.
(CSV)
