# Supplementary material for: Spatial distribution and associated factors influencing 2024 Measles-Rubella vaccination campaign coverage among children aged 9–59 months in Mainland Tanzania: A multi-level mixed-effect analysis
Source: PLoS One. 2026 Jul 21;21(7):e0330134. doi: 10.1371/journal.pone.0330134 (PMC13387559; doi:10.1371/journal.pone.0330134)
Supplement: Attachment — Submitted filename: Response to Reviewers.docx [file pone.0330134.s003.docx]

F33-LHD-v19.2

*Please, reply to:* hmsuya@ihi.or.tz

**Location:** DAR ES SALAAM

February 4, 2026

Academic Editor, PLOS One

| **Re:** | **Response to Reviewers – Manuscript ID PONE-D-25-27325 Title: Spatial Distribution and Associated Factors Influencing 2024 Measles-Rubella Vaccination Campaign Coverage among Children Aged 9–59 Months in Mainland Tanzania** |
| --- | --- |

**Dear Academic Editor and Reviewers,**

We sincerely thank the Academic Editor and Reviewer #1 for their meticulous review, insightful comments, and constructive recommendations, which have substantially strengthened the scientific rigor, clarity, and interpretive depth of our manuscript. We have carefully addressed all points raised and revised the manuscript accordingly. Below, we provide a detailed, point-by-point response to each comment.

**Reviewer #1 – Comments and Responses**

**Comment 1:** The necessary information on Moran's I estimation for autocorrelation analysis is missing.
**Response:** We have included a detailed description of the Moran’s I statistic, including its formula, interpretation, and rationale for assessing spatial autocorrelation of MR vaccination coverage. This addition is now presented in the methods section of the track changed manuscript (page 9, line 271-278).

**Comment 2:** What was the scale or level of aggregation for which autocorrelation was attempted?
**Response:** The analysis was conducted at the regional level, encompassing all 26 administrative regions of the Mainland Tanzania. This has been clarified in the revised methods section of the track changed manuscript. (Page 9, line 280)

**Comment 3:** How many sub-regions were there?
**Response:** A total of 1,483 sub-regions, represented as enumeration areas, were included in the spatial analysis for this study. This has been clarified in the revised methods section of the tracked-changes manuscript. (Page 9, line 280).

**Comment 4:** How were the neighbours defined—by contiguity, distance bands, or k-nearest?
**Response:** Neighbours relationships were defined using first-order queen contiguity, ensuring that each region’s immediate geographic neighbors were accurately incorporated into the spatial analysis. This has been clarified in the revised methods section of the tracked-changes manuscript. (Page 9, line 281-282)

**Comment 5:** What type of weight matrix was defined for the analysis?
**Response:** A row –standardized spatial weight matrix was applied consistently across all Moran’s I and Getis-Ord Gi* analyses. This has been clarified in the revised methods section of the tracked-changes manuscript. (Page 9, line 282-284)

**Comment 6:** Moran's I value of 0.34 needs to be compared and contrasted with similar health situations.
**Response:** In the discussion, we contextualize the observed Moran’s I of 0.34, highlighting it as indicative of moderate spatial clustering. Comparisons with prior studies on vaccination coverage and other public health outcomes in Sub-Saharan Africa are now included to demonstrate consistency and relevance. This has been clarified in the discussion section of the revised tracked-changes manuscript. (Page 27, line 526-532)

**Comment 7:** After global Moran's I, provide local Moran’s I (LISA) to identify outliers.
**Response:** We have incorporated local Moran’s (LISA) analysis to identify High-High clusters, Low-Low clusters, High-Low outliers, and Low-High outliers at regional and sub-regional levels. This has been clarified in the revised methods section of the tracked-changes manuscript (Page 9, line 284-286)

**Comment 8:** In the Getis-Ord Gi* analysis, include confidence limits in addition to z-values.
**Response:** We have incorporated the 90%, 95% and 99% confidence limits alongside z-scores for all Getis-Ord Gi* hot and cold spot analyses, improving the statistical robustness and interpretive reliability of the spatial clustering results. This has been clarified in the revised methods section of the tracked-changes manuscript. (Page 10, line 302-305)

**Comment 9:** If clustering was explored at different geographic scales, report any differences (MAUP).
**Response:** We explicitly address the Modifiable Areal Unit Problem (MAUP) in the methods and discussion sections. In the methods section this has been clarified in the revised tracked-changes manuscript. (Page 10, line 310-314). While in the discussion section it has been discussed on the revised tracked-changes manuscript. (Page 28, line 578-584)

**Comment 10:** Was spatial dependence accounted for in subsequent models?
**Response:** We did not consider the spatial regression approaches, including spatial lag and spatial error models in the methods and discussion sections. In the methods section this has been clarified in the revised tracked-changes manuscript. (Page 10, line 308-310) While in the discussion section it has been discussed on the revised tracked-changes manuscript. (Page 29, line 589-595)

**Comment 11:** The mention of Geospatial Artificial Intelligence is unclear.
**Response:** References to Geospatial Artificial Intelligence have been removed from the Abstract (Page 3, line 65-69) and Conclusion (Page 31, line 671-676), as they were not directly relevant to the current analyses.

**Comment 12:** State the specific R packages and software versions used.
**Response:** We have added the following details in the methods section (Page 11, line 325-327)

- **R version 4.3.1**
- **spdep** for Moran’s I and spatial weights
- **sf** and **rgdal** for spatial data handling
- **spatstat** and **GISTools** for hot spot analyses

**Comment 13:** Improve language clarity.
**Response:** We performed comprehensive language editing throughout the manuscript. For example, the original sentence: “The majority of the surveyed caregiver’s were married/cohabited. In term of wealth, 5621 (41.3%) of the vaccinated children during the campaign came from the middle (Table 1)” has been revised to: *“The majority of surveyed caregivers were married or cohabiting. Regarding wealth, 5,621 (41.3%) of vaccinated children during the campaign came from households with middle-level wealth (Table 1).”* (Page 11, line 351-353)

**Data Availability:** We confirm that all data required to replicate the findings are available upon the request from the corresponding author. The Data Availability Statement has been updated to reflect this. The minimal dataset required to reproduce the reported measures and generate the figures presented in the manuscript has been provided.

**Figures (Maps):** Figures 1 and 2 have been replaced with public-domain map sources (Natural Earth and USGS), fully compliant with CC BY 4.0 licensing. Figure captions have been updated to clearly indicate the sources and licensing, and the figures have been pre-flight-checked using PACE.

**Reviewer Suggested References:** We thank the reviewer for suggesting several references. After careful evaluation, relevant citations have been incorporated into the Introduction, Methods and Discussion to provide additional context and support [Reference numbers 31-34 on the Page 28, line 757-769, Reference numbers 43-55 on the Page 29, line 790-827]. References deemed not directly relevant to our study scope were not included.

**Closing Statement:** We sincerely appreciate the reviewer’s thoughtful critique and guidance. All suggestions have been carefully addressed to enhance methodological transparency, analytical rigor, and clarity of presentation. We are confident that these revisions substantially improve the manuscript and its contribution to understanding MR vaccination coverage and spatial disparities in Tanzania.

Thank you for your time and consideration.

Kind regards
**Hajirani Msuya, MSc**
(on behalf of all authors)
