# Supplementary material for: Spatial distribution and associated factors influencing 2024 Measles-Rubella vaccination campaign coverage among children aged 9–59 months in Mainland Tanzania: A multi-level mixed-effect analysis
Source: PLoS One. 2026 Jul 21;21(7):e0330134. doi: 10.1371/journal.pone.0330134 (PMC13387559; doi:10.1371/journal.pone.0330134)
Supplement: Attachment — Submitted filename: Response_to_reviewers.docx [file pone.0330134.s004.docx]

F33-LHD-v19.2

*Please, reply to:* hmsuya@ihi.or.tz

**Location:** DAR ES SALAAM

March 27, 2026

Academic Editor and Reviewers, PLOS One

| **Re:** | **Response to Reviewers – Manuscript ID: PONE-D-25-27325R1 Title: Spatial Distribution and Associated Factors Influencing 2024 Measles-Rubella Vaccination Campaign Coverage among Children Aged 9–59 Months in Mainland Tanzania** |
| --- | --- |

**Dear Academic Editor and Reviewers,**

We would like to sincerely thank you for the careful review of our manuscript and for the constructive and insightful comments. We appreciate the time and expertise invested in improving the scientific rigor and clarity of our work. We have carefully revised the manuscript to address all comments. Major revisions include refinement of the spatial statistical interpretation, use of standard significance thresholds, adoption of standard cartographic colour schemes, and clarification of LISA cluster definitions.

Below we provide a detailed point-by-point response.

**Response to Academic Editor comment**

**Comment:** *The statistical analysis seems over interpreted. There is no need for comparison of multiple CI values; a single statistically significant value such as 95% is sufficient.*

**Response:** We thank the editor for this important observation. We agree that using multiple confidence levels (90%, 95%, and 99%) may introduce unnecessary complexity and potentially over interpret the statistical results. We have therefore revised the analysis to use only the standard 95% significance threshold (z ≥ ±1.96), which is widely recommended in spatial epidemiology literature.

**Changes made:**

- Removed all references to 90% and 99% CI
- Reclassified hotspot analysis using only 95% significance
- Updated all figures and text accordingly
- Revised methods section to clearly describe the statistical threshold used

These changes appear in:

- **Abstract section** line numbers 29 – 33 of the track changed manuscript
- **Methods section (Spatial analysis subsection)** line numbers 254-262 of the track changed manuscript
- **Results section (Hotspot analysis subsection)** line number 396 – 410 of the track changed manuscript
- **Fig 3 - Corrected**

**Response to Editor comment on colour standards**

**Comment:** *There are some standard colours for maps and statistics that may help.*

**Response:** We appreciate this important suggestion. We have revised all spatial maps to follow standard spatial epidemiology cartographic conventions commonly used in LISA and hotspot analysis.

The revised colour scheme now follows standard practice:

- **Hotspots (High–High): red**
- **Cold spots (Low–Low): blue**
- **Low–High outliers: green**
- **High–Low outliers: light blue**
- **Not significant: pale cream**

**Changes made:**

- Updated all LISA and Gi* maps
- Updated figure legends
- Updated map descriptions in results
- Updated interpretation text

These revisions improve interpretability and align with standard geospatial visualization practices.

**Response to comment on LISA interpretation**

**Comment:** *There are questions about the significance of some statistical indicators like the local Moran and Geary indicator within the LISA system.*

**Response:** We thank the reviewer for highlighting this important methodological issue. We carefully reviewed the recommended reference (Chen, 2024) and revised our interpretation of LISA statistics accordingly.

Specifically, we clarified that:

- **High–High clusters represent hotspots** line number 264-265 of the track changed manuscript
- **Low–Low clusters represent cold spots** line number 265-267 of the track changed manuscript
- **High–Low and Low–High represent spatial outliers** line number 267-268 of the track changed manuscript. These outliers were not being interpreted as clusters line number 270-271 of the track changed manuscript

We also revised the description of Local Moran’s I interpretation to align with standard spatial statistics definitions.

**Changes made:**

- Revised LISA interpretation in Methods
- Corrected definitions of clusters and outliers
- Corrected interpretation errors noted by reviewer

These changes appear in:

- **Methods section** line number 264-271 of the track changed manuscript
- **Results section** line number 340 – 365 of the track changed manuscript

**Response to Reviewer #1**

**Comment:** *The manuscript will benefit from scrutiny by an expert in geostatistical analysis.*

**Response:** We appreciate this recommendation. Following this suggestion, we carefully reviewed the spatial statistical analysis and interpretation and revised the manuscript accordingly to improve methodological clarity and correctness. Key improvements include:

- Simplified statistical significance interpretation
- Corrected LISA cluster definitions
- Adoption of standard mapping conventions
- Improved explanation of spatial statistics

We believe these revisions have significantly strengthened the technical rigor of the manuscript.

**Comment:** *Why use 90%, 95%, and 99% CI values? One CI at 95% would be sufficient.*

**Response:** We agree with the reviewer. We have removed the multiple confidence thresholds and now use only the 95% significance level, which is standard practice in spatial epidemiology.

**Changes made:**

- Removed 90% and 99% significance categories
- Revised hotspot classification
- Updated figures and legends
- Updated statistical description

**Comment:** *There are factual errors (example: explanation of cold spots is wrong).*

**Response:** We thank the reviewer for identifying this issue. We have corrected the definition of cold spots. The revised definition now states: Cold spots represent areas with low values surrounded by neighbouring areas with similarly low values (Low–Low clusters). This correction has been made throughout the manuscript.

**Changes made:**

- Corrected definition in Results section line number 340 – 365 of the track changed manuscript
- Corrected Fig 2 and Fig 3 descriptions

**Comment:** *LISA maps should use standard colour schema and standard pattern.*

**Response:** We appreciate this suggestion and have updated all LISA maps to use standard spatial clustering colours commonly used in geostatistical literature.

**Changes made:**

- Updated LISA map colours
- Updated legends
- Updated figure captions
- Updated interpretation text

**Additional improvements made**

In addition to the reviewer comments, we also:

- Improved clarity of spatial statistical descriptions
- Simplified interpretation language
- Corrected minor typographical errors
- Improved figure readability
- Improved methodological transparency

**Conclusion:** We sincerely thank the Academic Editor and Reviewer for their constructive comments, which have substantially improved the quality, clarity, and scientific rigor of our manuscript. We believe the revisions have adequately addressed all concerns raised.

**Figures (Maps):** Figures 1 and 2 have been replaced with public-domain map sources (Natural Earth and USGS), fully compliant with CC BY 4.0 licensing. Figure captions have been updated to clearly indicate the sources and licensing, and the figures have been pre-flight-checked using PACE

**Closing Statement:** We sincerely appreciate the editors and reviewer’s thoughtful critique and guidance. All suggestions have been carefully addressed to enhance methodological transparency, analytical rigor, and clarity of presentation. We are confident that these revisions substantially improve the manuscript and its contribution to understanding MR vaccination coverage and spatial disparities in Tanzania.

Thank you for your time and consideration.

Sincerely,

**Hajirani M. Msuya, MSc**
Ifakara Health Institute
Dar es Salaam, Tanzania
Email: [hmsuya@ihi.or.tz](mailto:hmsuya@ihi.or.tz)

(On behalf of all co-authors)
