# Supplementary material for: Spatial distribution and associated factors influencing 2024 Measles-Rubella vaccination campaign coverage among children aged 9–59 months in Mainland Tanzania: A multi-level mixed-effect analysis
Source: PLoS One. 2026 Jul 21;21(7):e0330134. doi: 10.1371/journal.pone.0330134 (PMC13387559; doi:10.1371/journal.pone.0330134)
Supplement: Attachment — Submitted filename: Measle_Rubella_Response_to_reviewers.docx [file pone.0330134.s005.docx]

F33-LHD-v19.2

*Please, reply to:* hmsuya@ihi.or.tz

**Location:** DAR ES SALAAM

May 29, 2026

Academic Editor and Reviewers, PLOS One

| **Re:** | **Response to Reviewers – Manuscript ID: PONE-D-25-27325R1  Title: Spatial Distribution and Associated Factors Influencing 2024 Measles-Rubella Vaccination Campaign Coverage among Children Aged 9–59 Months in Mainland Tanzania: A multi-level mixed-effect analysis** |
| --- | --- |

Dear Dr. Pierre Roques and Reviewer,

We sincerely thank you for the careful review of our manuscript and for the constructive comments and suggestions. We appreciate the valuable feedback, which has helped us improve the clarity, methodological rigor, and presentation of the manuscript.

We have carefully revised the manuscript accordingly. All changes made in the revised manuscript are highlighted in yellow in the tracked-changes version. Below, we provide a detailed point-by-point response to each comment.

**Responses to the Academic Editor**

**Journal Requirements:**

**Comment 1:** *Please take into account the last suggestion from the reviewer specifically about the reference population used in the statistical analysis and perhaps the color used in the map (but this is really minor, just take care that it is explained in each of the figure legend). In addition, take care about the wording and cesure within the text as there are very few correction in the final step for publication.*

**Response:** Thank you for this valuable guidance. We carefully revised the methodology section and critically reassessed the cited references in response to the reviewer’s comments. Specifically, we refined the references and accompanying descriptions related to the classification of high vaccination coverage (favorable outcome) and low vaccination coverage (unfavorable outcome), as reflected in the revised track-changed manuscript (Page 7, Lines 215–226).

In addition, we reviewed and updated references related to previously published methodological work, including the citation of Yanguang Chen (2024) concerning Local Indicators of Spatial Association (LISA), which has been incorporated into the revised manuscript (Page 8, Lines 250–253).

Furthermore, the color scheme used in all maps and spatial figures was revised to improve interpretability and consistency with standard epidemiological visualization practices. Higher vaccination coverage is now represented using green color gradients, whereas lower coverage is represented using red color gradients. Corresponding clarifications have also been added to all figure legends to enhance readability and facilitate interpretation.

**Comment 2:** Please review your reference list to ensure that it is complete and correct. If you have cited papers that have been retracted, please include the rationale for doing so in the manuscript text, or remove these references and replace them with relevant current references. Any changes to the reference list should be mentioned in the rebuttal letter that accompanies your revised manuscript. If you need to cite a retracted article, indicate the article’s retracted status in the References list and also include a citation and full reference for the retraction notice.

**Response:** Thank you for this important comment. We carefully reviewed the entire reference list to ensure completeness, accuracy, and consistency with the journal guidelines. During this process, duplicate references were identified and removed, and bibliographic details were corrected and standardized where necessary.

In addition, all cited publications were screened for retraction status using publisher records and available retraction databases. No retracted articles were identified among the references cited in the revised manuscript; therefore, no retraction notices or corresponding justifications were required.

All revisions made to the reference list have been incorporated into the revised manuscript, with the corresponding changes highlighted in yellow for ease of identification.

**Responses to Reviewer #1**

We sincerely appreciate the reviewer’s positive evaluation and constructive recommendations.

**Comment 1:** *In Table 2, the ‘overall’ estimates could be placed as the last row.*

**Response:** Thank you for this helpful suggestion. We have revised Table 2 accordingly by relocating the “Overall” estimates to the final row of the table to enhance clarity, readability, and consistency in presentation. This revision is reflected in the updated manuscript (Page 13, Line 347) in the tracked-changes version.

**Comment 2:** *You are plotting MR vaccination coverage, so the colour schema of the maps should be reversed, high coverage (favourable outcome) should be given green and unfavorable outcome should be given ‘red’. This should be adapted in all maps.*

**Response:** Thank you for this helpful observation. We have revised the color scheme across all maps to improve interpretability and to align with conventional epidemiological visualization practices, whereby higher vaccination coverage is represented in green and lower coverage in red, reflecting favorable and unfavorable outcomes, respectively. This revision is documented in the methodology section of the revised tracked manuscript (Page 9, Lines 276–285).

In addition, the figure legends have been updated to explicitly describe the revised color gradients to ensure clarity and consistency in interpretation. These updates are reflected in the Results section (Page 14, Lines 367–381; and Page 15, Lines 393–398) of the revised tracked manuscript.

**Comment 3:** *The groups of marital status should be collapsed to ‘married/co-habitated’ and ‘others’ in the multivariate analysis. Similarly, the sex of the respondent can be omitted, as the vast majority (97.5%) belongs to one sex.*

**Response:** Thank you for this insightful recommendation. We have revised the multivariable analysis accordingly by collapsing the marital status categories into two groups, “Married/Cohabiting” and “Others,” in order to improve statistical stability and enhance interpretability of the results. Furthermore, sex of the respondent was excluded from the multivariable model due to limited variability in the sample, as the majority of respondents were female.

These revisions have been implemented throughout the manuscript, with corresponding updates reflected in Table 1 (Pages 11–12) and Table 3 (Pages 17–19) of the revised tracked manuscript.

**Comment 4:** *Reconsider the inclusion of regions in the multivariate analysis. Why is ‘Arusha’ province taken as the comparator? If you still wish to keep it, use “Dar Es Salaam”, and justify that being the capital city, it is used as the comparator.*

**Response:** Thank you for this valuable comment. We have re-evaluated the regional reference category and revised the multivariable analysis by setting Dar es Salaam as the reference category. This decision was based on its status as the largest urban and administrative center in the country, with comparatively better access to healthcare services and immunization infrastructure, thereby providing a meaningful benchmark for regional comparisons.

This justification has now been incorporated into the statistical analysis subsection of the Results section and is reflected in Table 3 (Pages 17–19) of the revised tracked manuscript.

**Comment 5:** *The following two sentences on page 9 could be modified…"In this study, the spatial regression models (e.g., spatial lag, spatial error, conditional autoregressive,
or geographically weighted regression) were not applied due to limited district- and ward-level
covariate data. Spatial autocorrelation analyses were performed at both regional and sub-region
levels to capture fine-scale variations in vaccination coverage and reduce aggregation bias from the
Modifiable Areal Unit Problem (MAUP) [32-34]," as*

*"**In this study, inferential spatial regression models were not fitted because adequate district- and ward-level covariate data were not available. Instead, spatial autocorrelation analyses were conducted at both regional and sub-regional administrative levels to examine spatial clustering in vaccination coverage and to explore potential scale-related effects, including those related to the Modifiable Areal Unit Problem (MAUP) [32–34]."*

**Response:** Thank you for the suggested wording improvement. We adopted the reviewer’s recommended wording with minor editorial adjustments for consistency. The revised text now reads:

“In this study, inferential spatial regression models were not fitted because adequate district- and ward-level covariate data were not available. Instead, spatial autocorrelation analyses were conducted at both regional and sub-regional administrative levels to examine spatial clustering in vaccination coverage and to explore potential scale-related effects, including those related to the Modifiable Areal Unit Problem (MAUP) [32–34].”

This revision enhances the clarity and precision of the description of the spatial analytical approach, as reflected in the revised manuscript (Page 9, Lines 287–293).

**Comment 6:** *Data availability concerns.*

**Response:** Thank you for highlighting this issue. We carefully reviewed the Data Availability Statement and revised it to ensure compliance with the PLOS ONE data sharing policy. The underlying dataset and supporting materials have now been provided as supplementary files/repository submission (modify according to your actual action), and the updated Data Availability Statement has been included in the revised manuscript.

**Comment 7:** *PLOS authors have the option to publish the peer review history of their article.*

**Response:** Thank you for the clarification. We understand that this option refers to the publication of reviewer comments, author responses, and editorial decision letters alongside the final published article to enhance transparency in the peer review process. We will indicate our preference during the submission process.

We once again thank the Academic Editor and Reviewer for their thoughtful comments and constructive suggestions. We believe that these revisions have substantially improved the quality and clarity of the manuscript, and we hope that the revised version will now be suitable for publication in PLOS ONE.

Sincerely,

**Hajirani M. Msuya**
(On behalf of all co-authors)
