# Supplementary material for: Spatial distribution and associated factors influencing 2024 Measles-Rubella vaccination campaign coverage among children aged 9–59 months in Mainland Tanzania: A multi-level mixed-effect analysis
Source: PLoS One. 2026 Jul 21;21(7):e0330134. doi: 10.1371/journal.pone.0330134 (PMC13387559; doi:10.1371/journal.pone.0330134)
Supplement: Peer review history

# Peer review: Decision Letter 0

**DOI:** 10.1371/journal.pone.0330134.r001

**Authors:** Pierre Roques

22 Sep 2025

PONE-D-25-27325

Spatial Distribution and Associated Factors Influencing 2024 Measles-Rubella Vaccination Campaign Coverage among Children Aged 9–59 Months in Mainland Tanzania

PLOS ONE

Dear Dr. Msuya,

Thank you for submitting your manuscript to PLOS ONE. After careful consideration, we feel that it has merit but does not fully meet PLOS ONE’s publication criteria as it currently stands. Therefore, we invite you to submit a revised version of the manuscript that addresses the points raised during the review process.

Specificaly please provide all the information about the statistical analysis you did as resquested by the reviewer.

Please submit your revised manuscript by Nov 06 2025 11:59PM. If you will need more time than this to complete your revisions, please reply to this message or contact the journal office at plosone@plos.org. When you're ready to submit your revision, log on to https://www.editorialmanager.com/pone/ and select the 'Submissions Needing Revision' folder to locate your manuscript file.

Please include the following items when submitting your revised manuscript:

- A rebuttal letter that responds to each point raised by the academic editor and reviewer(s). You should upload this letter as a separate file labeled 'Response to Reviewers'.
- A marked-up copy of your manuscript that highlights changes made to the original version. You should upload this as a separate file labeled 'Revised Manuscript with Track Changes'.
- An unmarked version of your revised paper without tracked changes. You should upload this as a separate file labeled 'Manuscript'.

If you would like to make changes to your financial disclosure, please include your updated statement in your cover letter. Guidelines for resubmitting your figure files are available below the reviewer comments at the end of this letter.

If applicable, we recommend that you deposit your laboratory protocols in protocols.io to enhance the reproducibility of your results. Protocols.io assigns your protocol its own identifier (DOI) so that it can be cited independently in the future. For instructions see: https://journals.plos.org/plosone/s/submission-guidelines#loc-laboratory-protocols. Additionally, PLOS ONE offers an option for publishing peer-reviewed Lab Protocol articles, which describe protocols hosted on protocols.io. Read more information on sharing protocols at https://plos.org/protocols?utm_medium=editorial-email&utm_source=authorletters&utm_campaign=protocols.

We look forward to receiving your revised manuscript.

Kind regards,

Pierre Roques, Ph.D.

Academic Editor

PLOS ONE

Journal Requirements:

When submitting your revision, we need you to address these additional requirements.

1.Please ensure that your manuscript meets PLOS ONE's style requirements, including those for file naming. The PLOS ONE style templates can be found at

https://journals.plos.org/plosone/s/file?id=wjVg/PLOSOne_formatting_sample_main_body.pdf and

https://journals.plos.org/plosone/s/file?id=ba62/PLOSOne_formatting_sample_title_authors_affiliations.pdf

2. Thank you for stating the following in your Competing Interests section:

“No”

Please complete your Competing Interests on the online submission form to state any Competing Interests. If you have no competing interests, please state "The authors have declared that no competing interests exist.", as detailed online in our guide for authors at http://journals.plos.org/plosone/s/submit-now

This information should be included in your cover letter; we will change the online submission form on your behalf.

3. We note that your Data Availability Statement is currently as follows: All relevant data are within the manuscript and in Supporting Information files.

Please confirm at this time whether or not your submission contains all raw data required to replicate the results of your study. Authors must share the “minimal data set” for their submission. PLOS defines the minimal data set to consist of the data required to replicate all study findings reported in the article, as well as related metadata and methods (https://journals.plos.org/plosone/s/data-availability#loc-minimal-data-set-definition).

For example, authors should submit the following data:

- The values behind the means, standard deviations and other measures reported;

- The values used to build graphs;

- The points extracted from images for analysis.

Authors do not need to submit their entire data set if only a portion of the data was used in the reported study.

If your submission does not contain these data, please either upload them as Supporting Information files or deposit them to a stable, public repository and provide us with the relevant URLs, DOIs, or accession numbers. For a list of recommended repositories, please see https://journals.plos.org/plosone/s/recommended-repositories.

If there are ethical or legal restrictions on sharing a de-identified data set, please explain them in detail (e.g., data contain potentially sensitive information, data are owned by a third-party organization, etc.) and who has imposed them (e.g., an ethics committee). Please also provide contact information for a data access committee, ethics committee, or other institutional body to which data requests may be sent. If data are owned by a third party, please indicate how others may request data access.

4. When completing the data availability statement of the submission form, you indicated that you will make your data available on acceptance. We strongly recommend all authors decide on a data sharing plan before acceptance, as the process can be lengthy and hold up publication timelines. Please note that, though access restrictions are acceptable now, your entire data will need to be made freely accessible if your manuscript is accepted for publication. This policy applies to all data except where public deposition would breach compliance with the protocol approved by your research ethics board. If you are unable to adhere to our open data policy, please kindly revise your statement to explain your reasoning and we will seek the editor's input on an exemption. Please be assured that, once you have provided your new statement, the assessment of your exemption will not hold up the peer review process.

5. We note that Figure 1 and 2 in your submission contain map/satellite images which may be copyrighted. All PLOS content is published under the Creative Commons Attribution License (CC BY 4.0), which means that the manuscript, images, and Supporting Information files will be freely available online, and any third party is permitted to access, download, copy, distribute, and use these materials in any way, even commercially, with proper attribution. For these reasons, we cannot publish previously copyrighted maps or satellite images created using proprietary data, such as Google software (Google Maps, Street View, and Earth). For more information, see our copyright guidelines: http://journals.plos.org/plosone/s/licenses-and-copyright.

We require you to either (1) present written permission from the copyright holder to publish these figures specifically under the CC BY 4.0 license, or (2) remove the figures from your submission:

1. You may seek permission from the original copyright holder of Figure 1 and 2  to publish the content specifically under the CC BY 4.0 license.

We recommend that you contact the original copyright holder with the Content Permission Form (http://journals.plos.org/plosone/s/file?id=7c09/content-permission-form.pdf) and the following text:

“I request permission for the open-access journal PLOS ONE to publish XXX under the Creative Commons Attribution License (CCAL) CC BY 4.0 (http://creativecommons.org/licenses/by/4.0/). Please be aware that this license allows unrestricted use and distribution, even commercially, by third parties. Please reply and provide explicit written permission to publish XXX under a CC BY license and complete the attached form.”

Please upload the completed Content Permission Form or other proof of granted permissions as an "Other" file with your submission.

In the figure caption of the copyrighted figure, please include the following text: “Reprinted from [ref] under a CC BY license, with permission from [name of publisher], original copyright [original copyright year].”

2. If you are unable to obtain permission from the original copyright holder to publish these figures under the CC BY 4.0 license or if the copyright holder’s requirements are incompatible with the CC BY 4.0 license, please either i) remove the figure or ii) supply a replacement figure that complies with the CC BY 4.0 license. Please check copyright information on all replacement figures and update the figure caption with source information. If applicable, please specify in the figure caption text when a figure is similar but not identical to the original image and is therefore for illustrative purposes only.

The following resources for replacing copyrighted map figures may be helpful:

USGS National Map Viewer (public domain): http://viewer.nationalmap.gov/viewer/

The Gateway to Astronaut Photography of Earth (public domain): http://eol.jsc.nasa.gov/sseop/clickmap/

Maps at the CIA (public domain): https://www.cia.gov/library/publications/the-world-factbook/index.html and https://www.cia.gov/library/publications/cia-maps-publications/index.html

NASA Earth Observatory (public domain): http://earthobservatory.nasa.gov/

Landsat: http://landsat.visibleearth.nasa.gov/

USGS EROS (Earth Resources Observatory and Science (EROS) Center) (public domain): http://eros.usgs.gov/#

Natural Earth (public domain): http://www.naturalearthdata.com/

6. If the reviewer comments include a recommendation to cite specific previously published works, please review and evaluate these publications to determine whether they are relevant and should be cited. There is no requirement to cite these works unless the editor has indicated otherwise.

Additional Editor Comments:

Reviewer #1: This is a good attempt in spatial exploration of the 2024 MR vaccination campaign coverage in Tanzania. There are a few concerns that can improve the readability and the uptake of vaccines in Tanzania.

1. The necessary information on Moran's I estimation for autocorrelation analysis are missing.

2. What was the scale or level of aggregation for which autocorrelation was attempted, was it at the regional, district, ward or any other sub-regional levels?

3. How many sub-regions were there?

4. How were the neighbours defined, by contiguity, distance bands or k-nearest?

5. What type of weight matrix was defined for the analysis?

6. Further in the discussion the Moran's I value of 0.34 for such/similar health situations needs to be compared and contrasted.

7. After global Moran's I estimation, we would naturally expect the authors to provide Mora's local estimation (LISA) to point out the outliers (the high-low and low-high regions, which are important input for health intervention.

8.. In case of hot spot analysis, the Gets-Ord Gi* analysis, too in addition to the z values, we would expect to see the confidence limits.

9. Fig.2 in the manuscript indicates that clustering was explored at different geographic scales. If the results varied across these scales, it would be important to report them explicitly, as such differences illustrate the Modifiable Areal Unit Problem (MAUP). Including this discussion would improve methodological transparency.

10. One major purpose of testing for spatial autocorrelation is to guide subsequent analyses that account for spatial dependence, like spatial lag or spatial error models, conditional autoregressive models, or geographically weighted regression, etc. I am curious to know if authors have attempted any of those, and if not, if the reasons for not doing.

11. There is a mention on Geospatial Artificial Intelligence, in the conclusion and even in the abstract. But I could not see any relationship to this article. It would be helpful, if the authors explain it or remove the mention.

12. It would be good to state the specific packages and the version of R software that they have used for specific analyses.

13. In general, the language is of acceptable quality, but there is still a scope for improvements, for example "The majority of the surveyed caregiver’s were married/cohabited. In term of wealth, 5621 (41.3%) of the vaccinated

children during the campaign came from the middle (Table 1)"

Thank you

[Note: HTML markup is below. Please do not edit.]

Reviewers' comments:

Reviewer's Responses to Questions

**Comments to the Author**

1. Is the manuscript technically sound, and do the data support the conclusions?

The manuscript must describe a technically sound piece of scientific research with data that supports the conclusions. Experiments must have been conducted rigorously, with appropriate controls, replication, and sample sizes. The conclusions must be drawn appropriately based on the data presented.

Reviewer #1: Yes

**********

2. Has the statistical analysis been performed appropriately and rigorously?

Reviewer #1: Yes

**********

3. Have the authors made all data underlying the findings in their manuscript fully available?

The PLOS Data policy requires authors to make all data underlying the findings described in their manuscript fully available without restriction, with rare exception (please refer to the Data Availability Statement in the manuscript PDF file). The data should be provided as part of the manuscript or its supporting information, or deposited to a public repository. For example, in addition to summary statistics, the data points behind means, medians and variance measures should be available. If there are restrictions on publicly sharing data—e.g. participant privacy or use of data from a third party—those must be specified.

Reviewer #1: No

**********

4. Is the manuscript presented in an intelligible fashion and written in standard English?

PLOS ONE does not copyedit accepted manuscripts, so the language in submitted articles must be clear, correct, and unambiguous. Any typographical or grammatical errors should be corrected at revision, so please note any specific errors here.

Reviewer #1: No

**********

5. Review Comments to the Author

Please use the space provided to explain your answers to the questions above. You may also include additional comments for the author, including concerns about dual publication, research ethics, or publication ethics. (Please upload your review as an attachment if it exceeds 20,000 characters)

Reviewer #1: This is a good attempt in spatial exploration of the 2024 MR vaccination campaign coverage in Tanzania. There are a few concerns that can improve the readability and the uptake of vaccines in Tanzania.

1. The necessary information on Moran's I estimation for autocorrelation analysis are missing.

2. What was the scale or level of aggregation for which autocorrelation was attempted, was it at the regional, district, ward or any other sub-regional levels?

3. How many sub-regions were there?

4. How were the neighbours defined, by contiguity, distance bands or k-nearest?

5. What type of weight matrix was defined for the analysis?

6. Further in the discussion the Moran's I value of 0.34 for such/similar health situations needs to be compared and contrasted.

7. After global Moran's I estimation, we would naturally expect the authors to provide Mora's local estimation (LISA) to point out the outliers (the high-low and low-high regions, which are important input for health intervention.

8.. In case of hot spot analysis, the Gets-Ord Gi* analysis, too in addition to the z values, we would expect to see the confidence limits.

9. Fig.2 in the manuscript indicates that clustering was explored at different geographic scales. If the results varied across these scales, it would be important to report them explicitly, as such differences illustrate the Modifiable Areal Unit Problem (MAUP). Including this discussion would improve methodological transparency.

10. One major purpose of testing for spatial autocorrelation is to guide subsequent analyses that account for spatial dependence, like spatial lag or spatial error models, conditional autoregressive models, or geographically weighted regression, etc. I am curious to know if authors have attempted any of those, and if not, if the reasons for not doing.

11. There is a mention on Geospatial Artificial Intelligence, in the conclusion and even in the abstract. But I could not see any relationship to this article. It would be helpful, if the authors explain it or remove the mention.

12. It would be good to state the specific packages and the version of R software that they have used for specific analyses.

13. In general, the language is of acceptable quality, but there is still a scope for improvements, for example "The majority of the surveyed caregiver’s were married/cohabited. In term of wealth, 5621 (41.3%) of the vaccinated

children during the campaign came from the middle (Table 1)"

Thank you

**********

6. PLOS authors have the option to publish the peer review history of their article (what does this mean?). If published, this will include your full peer review and any attached files.

If you choose “no”, your identity will remain anonymous but your review may still be made public.

**Do you want your identity to be public for this peer review?**  For information about this choice, including consent withdrawal, please see our Privacy Policy.

Reviewer #1: **Yes:** Biju Soman

**********

[NOTE: If reviewer comments were submitted as an attachment file, they will be attached to this email and accessible via the submission site. Please log into your account, locate the manuscript record, and check for the action link "View Attachments". If this link does not appear, there are no attachment files.]

While revising your submission, please upload your figure files to the Preflight Analysis and Conversion Engine (PACE) digital diagnostic tool, https://pacev2.apexcovantage.com/. PACE helps ensure that figures meet PLOS requirements. To use PACE, you must first register as a user. Registration is free. Then, login and navigate to the UPLOAD tab, where you will find detailed instructions on how to use the tool. If you encounter any issues or have any questions when using PACE, please email PLOS at figures@plos.org. Please note that Supporting Information files do not need this step.

# Author comment: Author response to Decision Letter 1

**DOI:** 10.1371/journal.pone.0330134.r002

27 Feb 2026

Dear Academic Editor and Reviewers,

We sincerely thank the Academic Editor and Reviewer #1 for their meticulous review, insightful comments, and constructive recommendations, which have substantially strengthened the scientific rigor, clarity, and interpretive depth of our manuscript. We have carefully addressed all points raised and revised the manuscript accordingly. Below, we provide a detailed, point-by-point response to each comment.

Reviewer #1 – Comments and Responses

Comment 1: The necessary information on Moran's I estimation for autocorrelation analysis is missing.

Response: We have included a detailed description of the Moran’s I statistic, including its formula, interpretation, and rationale for assessing spatial autocorrelation of MR vaccination coverage. This addition is now presented in the methods section of the track changed manuscript (page 9, line 271-278).

Comment 2: What was the scale or level of aggregation for which autocorrelation was attempted?

Response: The analysis was conducted at the regional level, encompassing all 26 administrative regions of the Mainland Tanzania. This has been clarified in the revised methods section of the track changed manuscript. (Page 9, line 280)

Comment 3: How many sub-regions were there?

Response: A total of 1,483 sub-regions, represented as enumeration areas, were included in the spatial analysis for this study. This has been clarified in the revised methods section of the tracked-changes manuscript. (Page 9, line 280).

Comment 4: How were the neighbours defined—by contiguity, distance bands, or k-nearest?

Response: Neighbours relationships were defined using first-order queen contiguity, ensuring that each region’s immediate geographic neighbors were accurately incorporated into the spatial analysis. This has been clarified in the revised methods section of the tracked-changes manuscript. (Page 9, line 281-282)

Comment 5: What type of weight matrix was defined for the analysis?

Response: A row –standardized spatial weight matrix was applied consistently across all Moran’s I and Getis-Ord Gi* analyses. This has been clarified in the revised methods section of the tracked-changes manuscript. (Page 9, line 282-284)

Comment 6: Moran's I value of 0.34 needs to be compared and contrasted with similar health situations.

Response: In the discussion, we contextualize the observed Moran’s I of 0.34, highlighting it as indicative of moderate spatial clustering. Comparisons with prior studies on vaccination coverage and other public health outcomes in Sub-Saharan Africa are now included to demonstrate consistency and relevance. This has been clarified in the discussion section of the revised tracked-changes manuscript. (Page 27, line 526-532)

Comment 7: After global Moran's I, provide local Moran’s I (LISA) to identify outliers.

Response: We have incorporated local Moran’s (LISA) analysis to identify High-High clusters, Low-Low clusters, High-Low outliers, and Low-High outliers at regional and sub-regional levels. This has been clarified in the revised methods section of the tracked-changes manuscript (Page 9, line 284-286)

Comment 8: In the Getis-Ord Gi* analysis, include confidence limits in addition to z-values.

Response: We have incorporated the 90%, 95% and 99% confidence limits alongside z-scores for all Getis-Ord Gi* hot and cold spot analyses, improving the statistical robustness and interpretive reliability of the spatial clustering results. This has been clarified in the revised methods section of the tracked-changes manuscript. (Page 10, line 302-305)

Comment 9: If clustering was explored at different geographic scales, report any differences (MAUP).

Response: We explicitly address the Modifiable Areal Unit Problem (MAUP) in the methods and discussion sections. In the methods section this has been clarified in the revised tracked-changes manuscript. (Page 10, line 310-314). While in the discussion section it has been discussed on the revised tracked-changes manuscript. (Page 28, line 578-584)

Comment 10: Was spatial dependence accounted for in subsequent models?

Response: We did not consider the spatial regression approaches, including spatial lag and spatial error models in the methods and discussion sections. In the methods section this has been clarified in the revised tracked-changes manuscript. (Page 10, line 308-310) While in the discussion section it has been discussed on the revised tracked-changes manuscript. (Page 29, line 589-595)

Comment 11: The mention of Geospatial Artificial Intelligence is unclear.

Response: References to Geospatial Artificial Intelligence have been removed from the Abstract (Page 3, line 65-69) and Conclusion (Page 31, line 671-676), as they were not directly relevant to the current analyses.

Comment 12: State the specific R packages and software versions used.

Response: We have added the following details in the methods section (Page 11, line 325-327)

• R version 4.3.1

• spdep for Moran’s I and spatial weights

• sf and rgdal for spatial data handling

• spatstat and GISTools for hot spot analyses

Comment 13: Improve language clarity.

Response: We performed comprehensive language editing throughout the manuscript. For example, the original sentence: “The majority of the surveyed caregiver’s were married/cohabited. In term of wealth, 5621 (41.3%) of the vaccinated children during the campaign came from the middle (Table 1)” has been revised to: “The majority of surveyed caregivers were married or cohabiting. Regarding wealth, 5,621 (41.3%) of vaccinated children during the campaign came from households with middle-level wealth (Table 1).” (Page 11, line 351-353)

Data Availability: We confirm that all data required to replicate the findings are available upon the request from the corresponding author. The Data Availability Statement has been updated to reflect this. The minimal dataset required to reproduce the reported measures and generate the figures presented in the manuscript has been provided.

Figures (Maps): Figures 1 and 2 have been replaced with public-domain map sources (Natural Earth and USGS), fully compliant with CC BY 4.0 licensing. Figure captions have been updated to clearly indicate the sources and licensing, and the figures have been pre-flight-checked using PACE.

Reviewer Suggested References: We thank the reviewer for suggesting several references. After careful evaluation, relevant citations have been incorporated into the Introduction, Methods and Discussion to provide additional context and support [Reference numbers 31-34 on the Page 28, line 757-769, Reference numbers 43-55 on the Page 29, line 790-827]. References deemed not directly relevant to our study scope were not included.

Closing Statement: We sincerely appreciate the reviewer’s thoughtful critique and guidance. All suggestions have been carefully addressed to enhance methodological transparency, analytical rigor, and clarity of presentation. We are confident that these revisions substantially improve the manuscript and its contribution to understanding MR vaccination coverage and spatial disparities in Tanzania.

Thank you for your time and consideration.

Kind regards

Hajirani Msuya, MSc

(on behalf of all authors)

Attachment Submitted filename: Response to Reviewers.docx

# Peer review: Decision Letter 1

**DOI:** 10.1371/journal.pone.0330134.r003

**Authors:** Pierre Roques

23 Mar 2026

PONE-D-25-27325R1

Spatial Distribution and Associated Factors Influencing 2024 Measles-Rubella Vaccination Campaign Coverage among Children Aged 9–59 Months in Mainland Tanzania

PLOS One

Dear Dr. Msuya,

Thank you for submitting your manuscript to PLOS ONE. After careful consideration, we feel that it has merit but does not fully meet PLOS ONE’s publication criteria as it currently stands. Therefore, we invite you to submit a revised version of the manuscript that addresses the points raised during the review process.

==============================

As noted by the reviewer, the statistical analysis seems to be overinterpreted, there is no need for comparison of multiple CI values, a single statisticaly significant value as 95% is sufficient. As noted there are some standar color for maps and stats that may help.

Perhaps the color should be not significant (pale cream), high (red), low-low (blue), low-high (green) and high-low (light blue). To note there is some questions about the significance of some statistical indicators like the local Moran and Geary indicator within the LISA (Local indicator of spatial association) system (see Y Chen PlosOne 2024 May 22;19(5):e0303456. doi: 10.1371/journal.pone.0303456).

In addition in my mind the hotspot in LISA are the are where are high values near high values and Cold spots (low values near low values) while the others are outliers (areas that differ from their neighbors.

probably a new rewording may help to take that comments in acount

==============================

Please submit your revised manuscript by May 07 2026 11:59PM. If you will need more time than this to complete your revisions, please reply to this message or contact the journal office at plosone@plos.org. When you're ready to submit your revision, log on to https://www.editorialmanager.com/pone/ and select the 'Submissions Needing Revision' folder to locate your manuscript file.

Please include the following items when submitting your revised manuscript:

- A letter that responds to each point raised by the academic editor and reviewer(s). You should upload this letter as a separate file labeled 'Response to Reviewers'.
- A marked-up copy of your manuscript that highlights changes made to the original version. You should upload this as a separate file labeled 'Revised Manuscript with Track Changes'.
- An unmarked version of your revised paper without tracked changes. You should upload this as a separate file labeled 'Manuscript'.

If you would like to make changes to your financial disclosure, please include your updated statement in your cover letter. Guidelines for resubmitting your figure files are available below the reviewer comments at the end of this letter.

If applicable, we recommend that you deposit your laboratory protocols in protocols.io to enhance the reproducibility of your results. Protocols.io assigns your protocol its own identifier (DOI) so that it can be cited independently in the future. For instructions see: https://journals.plos.org/plosone/s/submission-guidelines#loc-laboratory-protocols. Additionally, PLOS ONE offers an option for publishing peer-reviewed Lab Protocol articles, which describe protocols hosted on protocols.io. Read more information on sharing protocols at https://plos.org/protocols?utm_medium=editorial-email&utm_source=authorletters&utm_campaign=protocols.

We look forward to receiving your revised manuscript.

Kind regards,

Pierre Roques, Ph.D.

Academic Editor

PLOS One

Journal Requirements:

If the reviewer comments include a recommendation to cite specific previously published works, please review and evaluate these publications to determine whether they are relevant and should be cited. There is no requirement to cite these works unless the editor has indicated otherwise.

[Note: HTML markup is below. Please do not edit.]

Reviewers' comments:

Reviewer's Responses to Questions

**Comments to the Author**

1. If the authors have adequately addressed your comments raised in a previous round of review and you feel that this manuscript is now acceptable for publication, you may indicate that here to bypass the “Comments to the Author” section, enter your conflict of interest statement in the “Confidential to Editor” section, and submit your "Accept" recommendation.

Reviewer #1: All comments have been addressed

**********

2. Is the manuscript technically sound, and do the data support the conclusions?

The manuscript must describe a technically sound piece of scientific research with data that supports the conclusions. Experiments must have been conducted rigorously, with appropriate controls, replication, and sample sizes. The conclusions must be drawn appropriately based on the data presented.

Reviewer #1: Partly

**********

3. Has the statistical analysis been performed appropriately and rigorously?

Reviewer #1: No

**********

4. Have the authors made all data underlying the findings in their manuscript fully available?

The PLOS Data policy requires authors to make all data underlying the findings described in their manuscript fully available without restriction, with rare exception (please refer to the Data Availability Statement in the manuscript PDF file). The data should be provided as part of the manuscript or its supporting information, or deposited to a public repository. For example, in addition to summary statistics, the data points behind means, medians and variance measures should be available. If there are restrictions on publicly sharing data—e.g. participant privacy or use of data from a third party—those must be specified.

Reviewer #1: Yes

**********

5. Is the manuscript presented in an intelligible fashion and written in standard English?

PLOS ONE does not copyedit accepted manuscripts, so the language in submitted articles must be clear, correct, and unambiguous. Any typographical or grammatical errors should be corrected at revision, so please note any specific errors here.

Reviewer #1: Yes

**********

6. Review Comments to the Author

Please use the space provided to explain your answers to the questions above. You may also include additional comments for the author, including concerns about dual publication, research ethics, or publication ethics. (Please upload your review as an attachment if it exceeds 20,000 characters)

Reviewer #1: I thank the authors for the revised manuscript; it is much better than the earlier version. Still, it seems the manuscript will benefit from a thorough scrutiny by an expert in geostatistical analysis. The interpretation of LISA estimates and their statistics need correction. Why you go for 90% 95% and 99% CI values? I feel one CI at 95% level would be sufficient. There are a few factual errors in the manuscript in a couple of instances (eg: Line 362-365; the explanation of cod spots is wrong). The LISA maps should use the standard colour schema and standard pattern for depicting hotspots and cold stops, and significance levels.

**********

7. PLOS authors have the option to publish the peer review history of their article (what does this mean?). If published, this will include your full peer review and any attached files.

If you choose “no”, your identity will remain anonymous but your review may still be made public.

**Do you want your identity to be public for this peer review?**  For information about this choice, including consent withdrawal, please see our Privacy Policy.

Reviewer #1: **Yes:** Biju Soman

**********

[NOTE: If reviewer comments were submitted as an attachment file, they will be attached to this email and accessible via the submission site. Please log into your account, locate the manuscript record, and check for the action link "View Attachments". If this link does not appear, there are no attachment files.]

To ensure your figures meet our technical requirements, please review our figure guidelines: https://journals.plos.org/plosone/s/figures

You may also use PLOS’s free figure tool, NAAS, to help you prepare publication quality figures: https://journals.plos.org/plosone/s/figures#loc-tools-for-figure-preparation.

NAAS will assess whether your figures meet our technical requirements by comparing each figure against our figure specifications.

# Author comment: Author response to Decision Letter 2

**DOI:** 10.1371/journal.pone.0330134.r004

27 Mar 2026

Academic Editor and Reviewers, PLOS One

Re: Response to Reviewers – Manuscript ID: PONE-D-25-27325R1

Title: Spatial Distribution and Associated Factors Influencing 2024 Measles-Rubella Vaccination Campaign Coverage among Children Aged 9–59 Months in Mainland Tanzania

Dear Academic Editor and Reviewers,

We would like to sincerely thank you for the careful review of our manuscript and for the constructive and insightful comments. We appreciate the time and expertise invested in improving the scientific rigor and clarity of our work. We have carefully revised the manuscript to address all comments. Major revisions include refinement of the spatial statistical interpretation, use of standard significance thresholds, adoption of standard cartographic colour schemes, and clarification of LISA cluster definitions.

Below we provide a detailed point-by-point response.

Response to Academic Editor comment

Comment: The statistical analysis seems over interpreted. There is no need for comparison of multiple CI values; a single statistically significant value such as 95% is sufficient.

Response: We thank the editor for this important observation. We agree that using multiple confidence levels (90%, 95%, and 99%) may introduce unnecessary complexity and potentially over interpret the statistical results. We have therefore revised the analysis to use only the standard 95% significance threshold (z ≥ ±1.96), which is widely recommended in spatial epidemiology literature.

Changes made:

• Removed all references to 90% and 99% CI

• Reclassified hotspot analysis using only 95% significance

• Updated all figures and text accordingly

• Revised methods section to clearly describe the statistical threshold used

These changes appear in:

• Abstract section line numbers 29 – 33 of the track changed manuscript

• Methods section (Spatial analysis subsection) line numbers 254-262 of the track changed manuscript

• Results section (Hotspot analysis subsection) line number 396 – 410 of the track changed manuscript

• Fig 3 - Corrected

Response to Editor comment on colour standards

Comment: There are some standard colours for maps and statistics that may help.

Response: We appreciate this important suggestion. We have revised all spatial maps to follow standard spatial epidemiology cartographic conventions commonly used in LISA and hotspot analysis.

The revised colour scheme now follows standard practice:

• Hotspots (High–High): red

• Cold spots (Low–Low): blue

• Low–High outliers: green

• High–Low outliers: light blue

• Not significant: pale cream

Changes made:

• Updated all LISA and Gi* maps

• Updated figure legends

• Updated map descriptions in results

• Updated interpretation text

These revisions improve interpretability and align with standard geospatial visualization practices.

Response to comment on LISA interpretation

Comment: There are questions about the significance of some statistical indicators like the local Moran and Geary indicator within the LISA system.

Response: We thank the reviewer for highlighting this important methodological issue. We carefully reviewed the recommended reference (Chen, 2024) and revised our interpretation of LISA statistics accordingly.

Specifically, we clarified that:

• High–High clusters represent hotspots line number 264-265 of the track changed manuscript

• Low–Low clusters represent cold spots line number 265-267 of the track changed manuscript

• High–Low and Low–High represent spatial outliers line number 267-268 of the track changed manuscript. These outliers were not being interpreted as clusters line number 270-271 of the track changed manuscript

We also revised the description of Local Moran’s I interpretation to align with standard spatial statistics definitions.

Changes made:

• Revised LISA interpretation in Methods

• Corrected definitions of clusters and outliers

• Corrected interpretation errors noted by reviewer

These changes appear in:

• Methods section line number 264-271 of the track changed manuscript

• Results section line number 340 – 365 of the track changed manuscript

Response to Reviewer #1

Comment: The manuscript will benefit from scrutiny by an expert in geostatistical analysis.

Response: We appreciate this recommendation. Following this suggestion, we carefully reviewed the spatial statistical analysis and interpretation and revised the manuscript accordingly to improve methodological clarity and correctness. Key improvements include:

• Simplified statistical significance interpretation

• Corrected LISA cluster definitions

• Adoption of standard mapping conventions

• Improved explanation of spatial statistics

We believe these revisions have significantly strengthened the technical rigor of the manuscript.

Comment: Why use 90%, 95%, and 99% CI values? One CI at 95% would be sufficient.

Response: We agree with the reviewer. We have removed the multiple confidence thresholds and now use only the 95% significance level, which is standard practice in spatial epidemiology.

Changes made:

• Removed 90% and 99% significance categories

• Revised hotspot classification

• Updated figures and legends

• Updated statistical description

Comment: There are factual errors (example: explanation of cold spots is wrong).

Response: We thank the reviewer for identifying this issue. We have corrected the definition of cold spots. The revised definition now states: Cold spots represent areas with low values surrounded by neighbouring areas with similarly low values (Low–Low clusters). This correction has been made throughout the manuscript.

Changes made:

• Corrected definition in Results section line number 340 – 365 of the track changed manuscript

• Corrected Fig 2 and Fig 3 descriptions

Comment: LISA maps should use standard colour schema and standard pattern.

Response: We appreciate this suggestion and have updated all LISA maps to use standard spatial clustering colours commonly used in geostatistical literature.

Changes made:

• Updated LISA map colours

• Updated legends

• Updated figure captions

• Updated interpretation text

Additional improvements made

In addition to the reviewer comments, we also:

• Improved clarity of spatial statistical descriptions

• Simplified interpretation language

• Corrected minor typographical errors

• Improved figure readability

• Improved methodological transparency

Conclusion: We sincerely thank the Academic Editor and Reviewer for their constructive comments, which have substantially improved the quality, clarity, and scientific rigor of our manuscript. We believe the revisions have adequately addressed all concerns raised.

Figures (Maps): Figures 1 and 2 have been replaced with public-domain map sources (Natural Earth and USGS), fully compliant with CC BY 4.0 licensing. Figure captions have been updated to clearly indicate the sources and licensing, and the figures have been pre-flight-checked using PACE

Closing Statement: We sincerely appreciate the editors and reviewer’s thoughtful critique and guidance. All suggestions have been carefully addressed to enhance methodological transparency, analytical rigor, and clarity of presentation. We are confident that these revisions substantially improve the manuscript and its contribution to understanding MR vaccination coverage and spatial disparities in Tanzania.

Thank you for your time and consideration.

Sincerely,

Hajirani M. Msuya, MSc

Ifakara Health Institute

Dar es Salaam, Tanzania

Email: hmsuya@ihi.or.tz

(On behalf of all co-authors)

Attachment Submitted filename: Response_to_reviewers.docx

# Peer review: Decision Letter 2

**DOI:** 10.1371/journal.pone.0330134.r005

**Authors:** Pierre Roques

30 Apr 2026

PONE-D-25-27325R2

Spatial Distribution and Associated Factors Influencing 2024 Measles-Rubella Vaccination Campaign Coverage among Children Aged 9–59 Months in Mainland Tanzania

PLOS One

Dear Dr. Msuya,

Thank you for submitting your manuscript to PLOS ONE. After careful consideration, we feel that it has merit but does not fully meet PLOS ONE’s publication criteria as it currently stands. Therefore, we invite you to submit a revised version of the manuscript that addresses the points raised during the review process.

==============================

Please take in account the last suggestion from the reviewer specifically about the reference population used in the statistical analysis and perhaps the color used in the map (but this is really minor, just take care that it is explained in each of the figure legend; In addition, take care about the wording and cesure within the text as there are very few correction in the final step for publication,

==============================

Please submit your revised manuscript by Jun 14 2026 11:59PM. If you will need more time than this to complete your revisions, please reply to this message or contact the journal office at plosone@plos.org. When you're ready to submit your revision, log on to https://www.editorialmanager.com/pone/ and select the 'Submissions Needing Revision' folder to locate your manuscript file.

Please include the following items when submitting your revised manuscript:

- A letter that responds to each point raised by the academic editor and reviewer(s). You should upload this letter as a separate file labeled 'Response to Reviewers'.
- A marked-up copy of your manuscript that highlights changes made to the original version. You should upload this as a separate file labeled 'Revised Manuscript with Track Changes'.
- An unmarked version of your revised paper without tracked changes. You should upload this as a separate file labeled 'Manuscript'.

If you would like to make changes to your financial disclosure, please include your updated statement in your cover letter. Guidelines for resubmitting your figure files are available below the reviewer comments at the end of this letter.

If applicable, we recommend that you deposit your laboratory protocols in protocols.io to enhance the reproducibility of your results. Protocols.io assigns your protocol its own identifier (DOI) so that it can be cited independently in the future. For instructions see: https://journals.plos.org/plosone/s/submission-guidelines#loc-laboratory-protocols. Additionally, PLOS ONE offers an option for publishing peer-reviewed Lab Protocol articles, which describe protocols hosted on protocols.io. Read more information on sharing protocols at https://plos.org/protocols?utm_medium=editorial-email&utm_source=authorletters&utm_campaign=protocols.

As the corresponding author, your ORCID iD is verified in the submission system and will appear in the published article. PLOS supports the use of ORCID, and we encourage all coauthors to register for an ORCID iD and use it as well. Please encourage your coauthors to verify their ORCID iD within the submission system before final acceptance, as unverified ORCID iDs will not appear in the published article. *Only*  the individual author can complete the verification step; PLOS staff *cannot*  verify ORCID iDs on behalf of authors.

We look forward to receiving your revised manuscript.

Kind regards,

Pierre Roques, Ph.D.

Academic Editor

PLOS One

Journal Requirements:

1. If the reviewer comments include a recommendation to cite specific previously published works, please review and evaluate these publications to determine whether they are relevant and should be cited. There is no requirement to cite these works unless the editor has indicated otherwise.

2. Please review your reference list to ensure that it is complete and correct. If you have cited papers that have been retracted, please include the rationale for doing so in the manuscript text, or remove these references and replace them with relevant current references. Any changes to the reference list should be mentioned in the rebuttal letter that accompanies your revised manuscript. If you need to cite a retracted article, indicate the article’s retracted status in the References list and also include a citation and full reference for the retraction notice.

[Note: HTML markup is below. Please do not edit.]

Reviewers' comments:

Reviewer's Responses to Questions

**Comments to the Author**

1. If the authors have adequately addressed your comments raised in a previous round of review and you feel that this manuscript is now acceptable for publication, you may indicate that here to bypass the “Comments to the Author” section, enter your conflict of interest statement in the “Confidential to Editor” section, and submit your "Accept" recommendation.

Reviewer #1: All comments have been addressed

**********

2. Is the manuscript technically sound, and do the data support the conclusions?

The manuscript must describe a technically sound piece of scientific research with data that supports the conclusions. Experiments must have been conducted rigorously, with appropriate controls, replication, and sample sizes. The conclusions must be drawn appropriately based on the data presented.

Reviewer #1: Yes

**********

3. Has the statistical analysis been performed appropriately and rigorously?

Reviewer #1: Yes

**********

4. Have the authors made all data underlying the findings in their manuscript fully available?

The PLOS Data policy requires authors to make all data underlying the findings described in their manuscript fully available without restriction, with rare exception (please refer to the Data Availability Statement in the manuscript PDF file). The data should be provided as part of the manuscript or its supporting information, or deposited to a public repository. For example, in addition to summary statistics, the data points behind means, medians and variance measures should be available. If there are restrictions on publicly sharing data—e.g. participant privacy or use of data from a third party—those must be specified.

Reviewer #1: No

**********

5. Is the manuscript presented in an intelligible fashion and written in standard English?

PLOS ONE does not copyedit accepted manuscripts, so the language in submitted articles must be clear, correct, and unambiguous. Any typographical or grammatical errors should be corrected at revision, so please note any specific errors here.

Reviewer #1: Yes

**********

6. Review Comments to the Author

Please use the space provided to explain your answers to the questions above. You may also include additional comments for the author, including concerns about dual publication, research ethics, or publication ethics. (Please upload your review as an attachment if it exceeds 20,000 characters)

Reviewer #1: Thank you for addressing the concerns raised earlier. Still there are a couple of things that will improve the manuscript.

1. In Table 2, the 'overall' estimates could be placed as the last row.

2. You are plotting MR vaccination coverage, so the colour schema of the maps should be reversed, high coverage (favourable outcome) should be given green and unfavorable outcome should be given 'red'. This should be adapted in all maps.

3. The groups of marital status should be collapsed to 'married/co-habitated' and 'others' (it does not make sense to keep too many groups, with very low proportions) in the multivariate analysis. Similarly, the sex of the respondent can be omitted, as the vast majority (97.5%) belongs to one sex.

4. Reconsider the inclusion of regions in the multivariate analysis. Why is 'Arusha' province taken as the comparator? If you still wish you keep it, use "Dar Es Salaam", and justify that being the capital city, it is used as the comparator.

5. The following two sentence on page 9 could be modified,

"In this study, the spatial regression models (e.g., spatial lag, spatial error, conditional autoregressive,

or geographically weighted regression) were not applied due to limited district- and ward-level

covariate data. Spatial autocorrelation analyses were performed at both regional and sub-region

levels to capture fine-scale variations in vaccination coverage and reduce aggregation bias from the

Modifiable Areal Unit Problem (MAUP) [32-34]," as

"In this study, inferential spatial regression models were not fitted because adequate district- and ward-level covariate data were not available. Instead, spatial autocorrelation analyses were conducted at both regional and sub-regional administrative levels to examine spatial clustering in vaccination coverage and to explore potential scale-related effects, including those related to the Modifiable Areal Unit Problem (MAUP) [32–34]."

**********

7. PLOS authors have the option to publish the peer review history of their article (what does this mean?). If published, this will include your full peer review and any attached files.

If you choose “no”, your identity will remain anonymous but your review may still be made public.

**Do you want your identity to be public for this peer review?**  For information about this choice, including consent withdrawal, please see our Privacy Policy.

Reviewer #1: **Yes:** Biju Soman

**********

[NOTE: If reviewer comments were submitted as an attachment file, they will be attached to this email and accessible via the submission site. Please log into your account, locate the manuscript record, and check for the action link "View Attachments". If this link does not appear, there are no attachment files.]

To ensure your figures meet our technical requirements, please review our figure guidelines: https://journals.plos.org/plosone/s/figures

You may also use PLOS’s free figure tool, NAAS, to help you prepare publication quality figures: https://journals.plos.org/plosone/s/figures#loc-tools-for-figure-preparation.

NAAS will assess whether your figures meet our technical requirements by comparing each figure against our figure specifications.

# Author comment: Author response to Decision Letter 3

**DOI:** 10.1371/journal.pone.0330134.r006

29 May 2026

Response to Reviewers – Manuscript ID: PONE-D-25-27325R1

Title: Spatial Distribution and Associated Factors Influencing 2024 Measles-Rubella Vaccination Campaign Coverage among Children Aged 9–59 Months in Mainland Tanzania: A multi-level mixed-effect analysis

Dear Dr. Pierre Roques and Reviewer,

We sincerely thank you for the careful review of our manuscript and for the constructive comments and suggestions. We appreciate the valuable feedback, which has helped us improve the clarity, methodological rigor, and presentation of the manuscript.

We have carefully revised the manuscript accordingly. All changes made in the revised manuscript are highlighted in yellow in the tracked-changes version. Below, we provide a detailed point-by-point response to each comment.

Responses to the Academic Editor

Journal Requirements:

Comment 1: Please take into account the last suggestion from the reviewer specifically about the reference population used in the statistical analysis and perhaps the color used in the map (but this is really minor, just take care that it is explained in each of the figure legend). In addition, take care about the wording and cesure within the text as there are very few correction in the final step for publication.

Response: Thank you for this valuable guidance. We carefully revised the methodology section and critically reassessed the cited references in response to the reviewer’s comments. Specifically, we refined the references and accompanying descriptions related to the classification of high vaccination coverage (favorable outcome) and low vaccination coverage (unfavorable outcome), as reflected in the revised track-changed manuscript (Page 7, Lines 215–226).

In addition, we reviewed and updated references related to previously published methodological work, including the citation of Yanguang Chen (2024) concerning Local Indicators of Spatial Association (LISA), which has been incorporated into the revised manuscript (Page 8, Lines 250–253).

Furthermore, the color scheme used in all maps and spatial figures was revised to improve interpretability and consistency with standard epidemiological visualization practices. Higher vaccination coverage is now represented using green color gradients, whereas lower coverage is represented using red color gradients. Corresponding clarifications have also been added to all figure legends to enhance readability and facilitate interpretation.

Comment 2: Please review your reference list to ensure that it is complete and correct. If you have cited papers that have been retracted, please include the rationale for doing so in the manuscript text, or remove these references and replace them with relevant current references. Any changes to the reference list should be mentioned in the rebuttal letter that accompanies your revised manuscript. If you need to cite a retracted article, indicate the article’s retracted status in the References list and also include a citation and full reference for the retraction notice.

Response: Thank you for this important comment. We carefully reviewed the entire reference list to ensure completeness, accuracy, and consistency with the journal guidelines. During this process, duplicate references were identified and removed, and bibliographic details were corrected and standardized where necessary.

In addition, all cited publications were screened for retraction status using publisher records and available retraction databases. No retracted articles were identified among the references cited in the revised manuscript; therefore, no retraction notices or corresponding justifications were required.

All revisions made to the reference list have been incorporated into the revised manuscript, with the corresponding changes highlighted in yellow for ease of identification.

Responses to Reviewer #1

We sincerely appreciate the reviewer’s positive evaluation and constructive recommendations.

Comment 1: In Table 2, the ‘overall’ estimates could be placed as the last row.

Response: Thank you for this helpful suggestion. We have revised Table 2 accordingly by relocating the “Overall” estimates to the final row of the table to enhance clarity, readability, and consistency in presentation. This revision is reflected in the updated manuscript (Page 13, Line 347) in the tracked-changes version.

Comment 2: You are plotting MR vaccination coverage, so the colour schema of the maps should be reversed, high coverage (favourable outcome) should be given green and unfavorable outcome should be given ‘red’. This should be adapted in all maps.

Response: Thank you for this helpful observation. We have revised the color scheme across all maps to improve interpretability and to align with conventional epidemiological visualization practices, whereby higher vaccination coverage is represented in green and lower coverage in red, reflecting favorable and unfavorable outcomes, respectively. This revision is documented in the methodology section of the revised tracked manuscript (Page 9, Lines 276–285).

In addition, the figure legends have been updated to explicitly describe the revised color gradients to ensure clarity and consistency in interpretation. These updates are reflected in the Results section (Page 14, Lines 367–381; and Page 15, Lines 393–398) of the revised tracked manuscript.

Comment 3: The groups of marital status should be collapsed to ‘married/co-habitated’ and ‘others’ in the multivariate analysis. Similarly, the sex of the respondent can be omitted, as the vast majority (97.5%) belongs to one sex.

Response: Thank you for this insightful recommendation. We have revised the multivariable analysis accordingly by collapsing the marital status categories into two groups, “Married/Cohabiting” and “Others,” in order to improve statistical stability and enhance interpretability of the results. Furthermore, sex of the respondent was excluded from the multivariable model due to limited variability in the sample, as the majority of respondents were female.

These revisions have been implemented throughout the manuscript, with corresponding updates reflected in Table 1 (Pages 11–12) and Table 3 (Pages 17–19) of the revised tracked manuscript.

Comment 4: Reconsider the inclusion of regions in the multivariate analysis. Why is ‘Arusha’ province taken as the comparator? If you still wish to keep it, use “Dar Es Salaam”, and justify that being the capital city, it is used as the comparator.

Response: Thank you for this valuable comment. We have re-evaluated the regional reference category and revised the multivariable analysis by setting Dar es Salaam as the reference category. This decision was based on its status as the largest urban and administrative center in the country, with comparatively better access to healthcare services and immunization infrastructure, thereby providing a meaningful benchmark for regional comparisons.

This justification has now been incorporated into the statistical analysis subsection of the Results section and is reflected in Table 3 (Pages 17–19) of the revised tracked manuscript.

Comment 5: The following two sentences on page 9 could be modified…"In this study, the spatial regression models (e.g., spatial lag, spatial error, conditional autoregressive,

or geographically weighted regression) were not applied due to limited district- and ward-level

covariate data. Spatial autocorrelation analyses were performed at both regional and sub-region

levels to capture fine-scale variations in vaccination coverage and reduce aggregation bias from the

Modifiable Areal Unit Problem (MAUP) [32-34]," as

"In this study, inferential spatial regression models were not fitted because adequate district- and ward-level covariate data were not available. Instead, spatial autocorrelation analyses were conducted at both regional and sub-regional administrative levels to examine spatial clustering in vaccination coverage and to explore potential scale-related effects, including those related to the Modifiable Areal Unit Problem (MAUP) [32–34]."

Response: Thank you for the suggested wording improvement. We adopted the reviewer’s recommended wording with minor editorial adjustments for consistency. The revised text now reads:

“In this study, inferential spatial regression models were not fitted because adequate district- and ward-level covariate data were not available. Instead, spatial autocorrelation analyses were conducted at both regional and sub-regional administrative levels to examine spatial clustering in vaccination coverage and to explore potential scale-related effects, including those related to the Modifiable Areal Unit Problem (MAUP) [32–34].”

This revision enhances the clarity and precision of the description of the spatial analytical approach, as reflected in the revised manuscript (Page 9, Lines 287–293).

Comment 6: Data availability concerns.

Response: Thank you for highlighting this issue. We carefully reviewed the Data Availability Statement and revised it to ensure compliance with the PLOS ONE data sharing policy. The underlying dataset and supporting materials have now been provided as supplementary files/repository submission (modify according to your actual action), and the updated Data Availability Statement has been included in the revised manuscript.

Comment 7: PLOS authors have the option to publish the peer review history of their article.

Response: Thank you for the clarification. We understand that this option refers to the publication of reviewer comments, author responses, and editorial decision letters alongside the final published article to enhance transparency in the peer review process. We will indicate our preference during the submission process.

We once again thank the Academic Editor and Reviewer for their thoughtful comments and constructive suggestions. We believe that these revisions have substantially improved the quality and clarity of the manuscript, and we hope that the revised version will now be suitable for publication in PLOS ONE.

Sincerely,

Hajirani M. Msuya

(On behalf of all co-authors)

Attachment Submitted filename: Measle_Rubella_Response_to_reviewers.docx

# Editor report: Decision Letter 3

**DOI:** 10.1371/journal.pone.0330134.r007

**Authors:** Pierre Roques

1 Jun 2026

Spatial Distribution and Associated Factors Influencing 2024 Measles-Rubella Vaccination Campaign Coverage among Children Aged 9–59 Months in Mainland Tanzania: A multi-level mixed-effect analysis

PONE-D-25-27325R3

Dear Dr. Msuya,

We’re pleased to inform you that your manuscript has been judged scientifically suitable for publication and will be formally accepted for publication once it meets all outstanding technical requirements.

Within one week, you’ll receive an e-mail detailing the required amendments. When these have been addressed, you’ll receive a formal acceptance letter and your manuscript will be scheduled for publication.

An invoice will be generated when your article is formally accepted. Please note, if your institution has a publishing partnership with PLOS and your article meets the relevant criteria, all or part of your publication costs will be covered. Please make sure your user information is up-to-date by logging into Editorial Manager at Editorial Manager® and clicking the ‘Update My Information' link at the top of the page. For questions related to billing, please contact billing support.

If your institution or institutions have a press office, please notify them about your upcoming paper to help maximize its impact. If they’ll be preparing press materials, please inform our press team as soon as possible -- no later than 48 hours after receiving the formal acceptance. Your manuscript will remain under strict press embargo until 2 pm Eastern Time on the date of publication. For more information, please contact onepress@plos.org.

Kind regards,

Pierre Roques, Ph.D.

Academic Editor

PLOS One

Additional Editor Comments (optional):

Reviewers' comments:

# Editor report: Acceptance letter

**DOI:** 10.1371/journal.pone.0330134.r008

**Authors:** Pierre Roques

PONE-D-25-27325R3

PLOS One

Dear Dr. Msuya,

I'm pleased to inform you that your manuscript has been deemed suitable for publication in PLOS One. Congratulations! Your manuscript is now being handed over to our production team.

At this stage, our production department will prepare your paper for publication. This includes ensuring the following:

* All references, tables, and figures are properly cited

* All relevant supporting information is included in the manuscript submission,

* There are no issues that prevent the paper from being properly typeset

You will receive further instructions from the production team, including instructions on how to review your proof when it is ready. Please keep in mind that we are working through a large volume of accepted articles, so please give us a few days to review your paper and let you know the next and final steps.

Lastly, if your institution or institutions have a press office, please let them know about your upcoming paper now to help maximize its impact. If they'll be preparing press materials, please inform our press team within the next 48 hours. Your manuscript will remain under strict press embargo until 2 pm Eastern Time on the date of publication. For more information, please contact onepress@plos.org.

You will receive an invoice from PLOS for your publication fee after your manuscript has reached the completed accept phase. If you receive an email requesting payment before acceptance or for any other service, this may be a phishing scheme. Learn how to identify phishing emails and protect your accounts at https://explore.plos.org/phishing.

If we can help with anything else, please email us at customercare@plos.org.

Thank you for submitting your work to PLOS ONE and supporting open access.

Kind regards,

PLOS ONE Editorial Office Staff

on behalf of

Dr. Pierre Roques

Academic Editor

PLOS One
